# *In Situ* Capture and Real-Time Enrichment of Marine Chemical Diversity

**DOI:** 10.1021/acscentsci.3c00661

**Published:** 2023-11-08

**Authors:** Morgane Mauduit, Marie Derrien, Marie Grenier, Stéphane Greff, Sacha Molinari, Pierre Chevaldonné, Charlotte Simmler, Thierry Pérez

**Affiliations:** IMBE, UMR CNRS 7263, IRD 237, Aix Marseille Université, Avignon Université, Station Marine d’Endoume, Chemin de la batterie des lions, 13007 Marseille, France

## Abstract

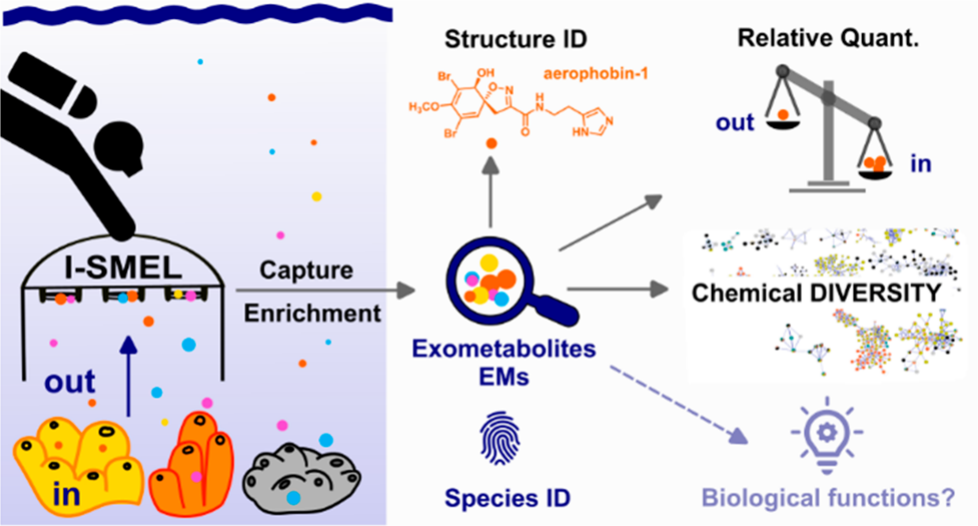

Analyzing the chemical composition of seawater to understand its influence on ecosystem functions is a long-lasting challenge due to the inherent complexity and dynamic nature of marine environments. Describing the intricate chemistry of seawater requires optimal *in situ* sampling. Here is presented a novel underwater hand-held solid-phase extraction device, I-SMEL (In Situ Marine moleculELogger), which aims to concentrate diluted molecules from large volumes of seawater in a delimited zone targeting keystone benthic species. Marine benthic holobionts, such as sponges, can impact the chemical composition of their surroundings possibly through the production and release of their specialized metabolites, hence termed exometabolites (EMs). I-SMEL was deployed in a sponge-dominated Mediterranean ecosystem at a 15 m depth. Untargeted MS-based metabolomics was performed on enriched EM extracts and showed (1) the chemical diversity of enriched seawater metabolites and (2) reproducible recovery and enrichment of specialized sponge EMs such as aerothionin, demethylfurospongin-4, and longamide B methyl ester. These EMs constitute the chemical identity of each targeted species: *Aplysina cavernicola*, *Spongia officinalis*, and *Agelas oroides*, respectively. I-SMEL concentrated sponge EMs from 10 L of water in a 10 min sampling time. The present proof of concept with I-SMEL opens new research perspectives in marine chemical ecology and sets the stage for further sustainable efforts in natural product chemistry.

## Abstract 2

I-SMEL chamber facilitates *in situ* capture and enrichment of waterborne molecules, thereby advancing investigations in marine chemical ecology and offering new prospects in natural product chemistry.

## Introduction

What lies in a drop of seawater? To the analytical chemist or marine ecologist, the answer is an ocean of molecules of diverse structures and origins, all diluted in trace quantities. In 1965, Wangersky wrote, “Seawater is a medium of a complexity sufficient to dismay any right-thinking analytical chemist”.^1^ Nevertheless, ocean scientists have tirelessly tried to unravel its complex nature, measuring how it has changed and interacted with the biosphere since the first life forms appeared on earth.^2^ Specialized sensors have been developed for the detection of known chemicals, whether they be natural or xenobiotic.^3^ Sample preparation has also evolved to capture diluted (e.g., emerging pollutants) and unknown molecules in aquatic environments using solid-phase extraction (SPE) devices.^4−6^ Such seawater enrichment led to complex extracts containing a wide and dynamic range of structurally diverse molecules. Advances in analytical chemistry enabled better chromatographic resolution with more sensitive and accurate mass spectrometry detection.^5,7^ As such, untargeted liquid chromatography tandem mass spectrometry (MS^2^), with the democratized use of open access software,^8^ led to the high-throughput detection of thousands of molecular signals, yielding dense metabolomic data sets interpreted with bioinformatic tools such as those in the Global Natural Products Social Molecular Networking systems (GNPS).^9^ When combined with *in silico*-based spectral annotation, these tools enable a deeper description of dense molecular data sets increasing the possible number of characterized chemical features.^10−12^ In marine ecology, such metabolomic approaches are deployed to study the composition of dissolved organic matter (DOM), a complex chemical mixture partially derived from metabolic activities of micro- and macro-organisms. Consequently, the field of marine metabolomics has expanded in environmental sciences, marine ecology,^13−15^ and natural product chemistry.^16,17^ Nevertheless, molecules diluted in seawater remain challenging to identify accurately due to (1) their unknown or multiple biosynthetic origins impeding taxonomical guidance for structural assignment, (2) their possible (bio)transformations, (3) the paucity of available commercial standards or related spectral data in open access libraries, and (4) their low abundance in collected samples precluding any purification. To date, Wangersky’s statement stands because identifying unknown molecules from seawater remains a daunting task.

Benthic marine holobionts are known to produce a plethora of structurally diverse specialized metabolites whose structural class and relative proportions are often taxonomically determined. These molecules contribute to the adaptation of an organism within an ecosystem, being involved in its defensive, growth, and communication strategies. Through their metabolic activities and cellular renewal, marine organisms may release part of their metabolites, hence termed exometabolites (EMs),^18,19^ which, either dissolved or bound to particles (e.g., cellular debris), may serve as nutrients and/or may be perceived as cues (i.e., allelochemicals sustaining communication between marine organisms).^20^ Deciphering chemical signatures within a pool of marine molecules requires studying the discrete metabolic contributions of marine organisms exuding their metabolites.^15,21^ However, once released, individual marine EMs are added to a pool of other chemicals, defining complex chemical seascapes equivalent to busy highways of putative allelochemicals. In light of the multiple transformations that diluted molecules may undergo in seawater, the composition of marine chemical seascapes is constantly changing in time and space.^20,21^ Consequently, techniques that enable immediate capture of molecules upon their release before extended biotransformations occur are critical to better understand the origins of such molecules and their functional roles. Such techniques would greatly support ecologists in their quest to identify key molecules that structure communication between organisms, thus providing arguments for the preservation of marine ecosystem functions. The structural diversity of marine specialized metabolites has also inspired drug discovery efforts. However, the global concern for biological resources management and biodiversity conservation^22^ has significantly slowed down natural product chemistry in the past, whereas attempts to cultivate marine organisms have not provided sustainable biosourcing yet. Therefore, the idea of alternative SPE approaches to sustainably access marine specialized EMs under natural conditions emerged^23−28^ with the objective of trapping the molecules of interest without harvesting the producing organisms.

**Figure 1:**
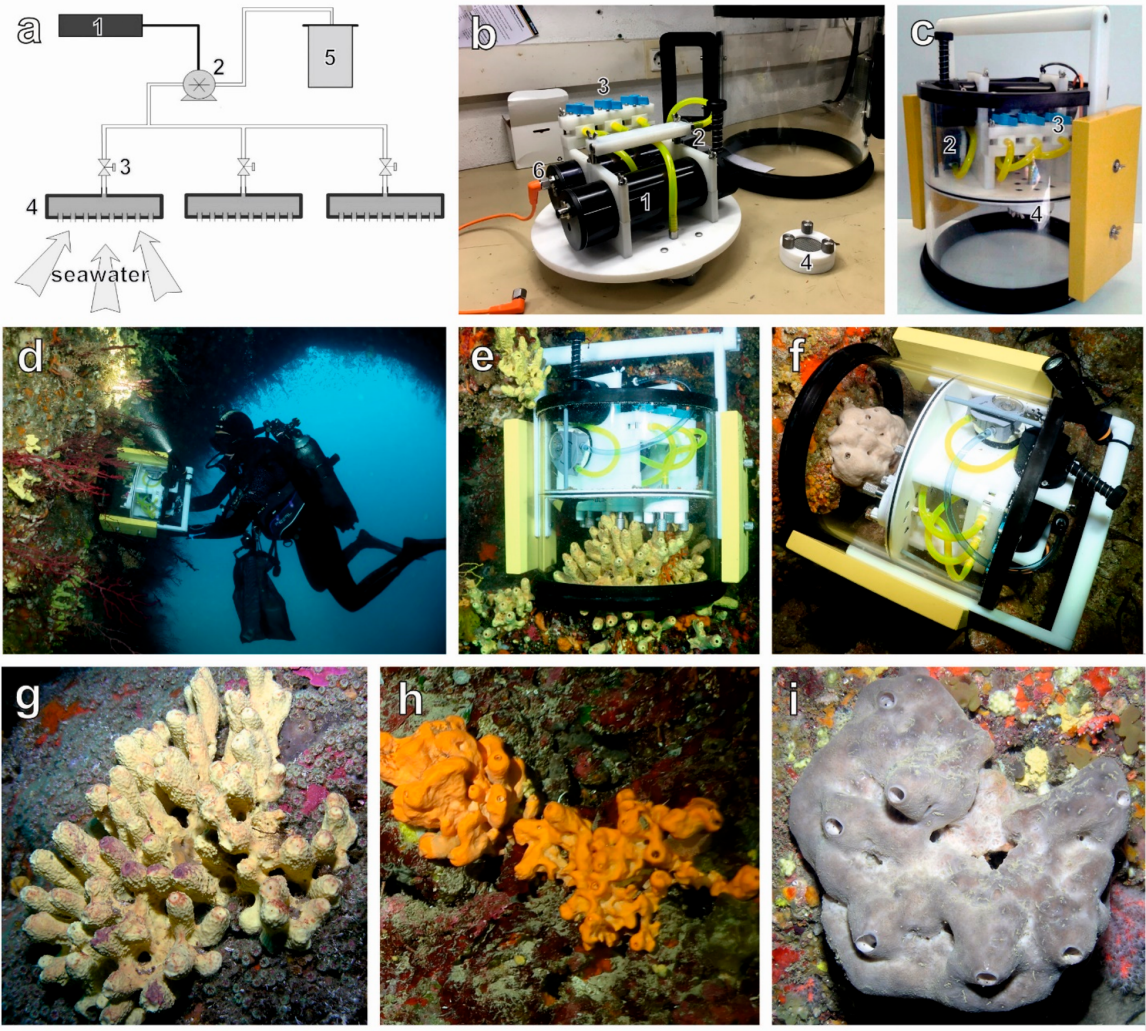
Representation of the I-SMEL instrument and its deployment in a Mediterranean marine ecosystem. Schematic view of the main functioning items (a) and views of the disassembled (b) and assembled (c) instrument (1. electronic controller of the pump, 2. peristaltic pump, 3. valves, 4. SPE ports, 5. pump outlet to connect a flexible tank, and 6. battery). See also S1 for more details. I-SMEL operated by a SCUBA diver in a coralligenous community (d) and then above the studied sponges (e, f). *In situ* pictures of the three targeted sponge species, *Aplysina cavernicola* (g), *Agelas oroides* (h), and *Spongia officinalis* (i).

**Figure 2:**
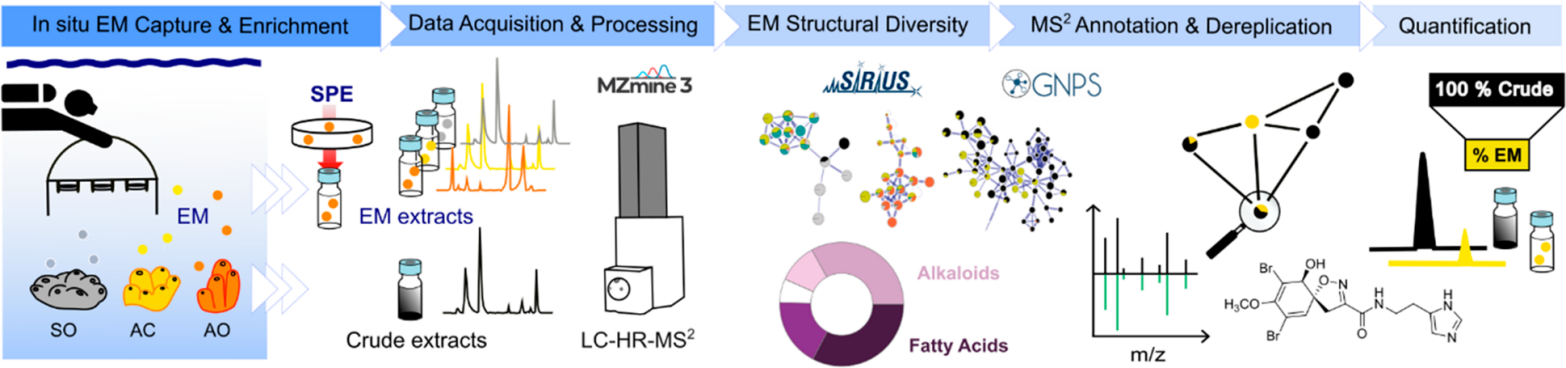
Sample preparation and analytical pipeline to characterize the structural diversity of captured marine exometabolites (EMs) and determine the proportion of sponge-specialized EMs. SO: *Spongia officinalis*. AC: *Aplysina cavernicola*. AO: *Agelas oroides*. HR-MS^2^: High-resolution tandem mass spectrometry. SPE: Solid-phase extraction.

The present work aims at evaluating the ability of a hand-held underwater SPE instrument to rapidly capture molecules within chemical seascapes and enrich specialized EMs released by keystone marine species in their ecosystems. This instrument has been named I-SMEL (In Situ Marine moleculELogger) (Figure 1). It was deployed and tested in the coralligenous, a Mediterranean ecosystem similar in many ways to tropical coral reefs in terms of species richness and functionality. The shaded parts of this ecosystem are made up of species assemblages dominated by sponges, which are prolific producers of specialized metabolites.^29,30^ Our experiments targeted three common and rather large species, whose specialized metabolites are well described in the literature, namely, *Aplysina cavernicola* (Vacelet, 1959), *Agelas oroides* (Schmidt, 1864), and *Spongia officinalis* (Linneus, 1759).^19,31−34^ Following *in situ* sampling and building on previous work,^15,19,35^ our analytical workflow (Figure 2) combines untargeted MS-based metabolomics, a description of molecular diversity using molecular class predictions with CANOPUS,^36,37^ and metabolite dereplication through molecular networking.^9,38,39^

## Results

### Presentation of the Instrument

I-SMEL was designed to (1) be easily handled underwater by a single user at different depths accessible by SCUBA diving, (2) facilitate the enrichment of molecules by filtering large (>10 L) volumes of seawater in a delimited zone within a short time (10–30 min), and (3) be versatile in its use depending on the configuration of the ecosystem to be sampled. The instrument is made of six primary components organized in two equal parts (Figure 1a–c, S1). The upper one encloses all of the electronics (peristaltic pump and its controller), tubing, and valves. The lower part is the 10 L chamber to be placed above the targeted benthic community or species (Figure 1d–f). Three separate supports for the SPE disk are fixed on the top of this chamber and can be replaced underwater (S1). They are individually connected to the peristaltic pump via independent valves, allowing three *in situ* captures either simultaneously or sequentially. I-SMEL can be placed in the water column, on horizontal bottoms, overhangs, or walls of different inclinations, and even on the ceiling of underwater caves. Once in place, seawater filtration starts by activating the pump with a simple push button at a defined duration and flow rate (here 1 min at 1 L/min), preset before the dive. SPE disks were preferred over more widely used cartridges as they are more appropriate when the water to be filtered is charged with different types of particulates. They also offer a larger surface of exchange, allowing faster flow rates to be applied, compatible with shorter sampling time. The disks, herein used, were made of one layer of glass fiber to retain particles and one layer of divinylbenzene polymer (DVB) to adsorb dissolved metabolites. DVB was chosen due to its demonstrated capacity to adsorb a wide range of nonpolar and semipolar DOM or specialized EMs.^40,41^

### *In Situ* Experiments

Three types of underwater experiments were performed. In the first type (EXP1), I-SMEL was deployed in the coralligenous ecosystem without targeting any benthic organism in particular. EXP1 aimed at concentrating molecules defining an average chemical seascape (ACS) of the target ecosystem. Three replicate captures were performed randomly by cumulatively filtering 10 L of water over five different communities. In the next experiments (EXP2-3), I-SMEL was placed above the targeted sponge species: *Aplysina cavernicola*, *Spongia officinalis*, and *Agelas oroides*. The objective of EXP2 was to concentrate sponge-specialized EMs on the SPE disks and thus evaluate whether I-SMEL could capture and enrich any EM if released by sponges. Each collection replicate of EXP2 accumulated a total of 10 L filtered on 3 DVB disks simultaneously by moving over five different sponge individuals. EXP3 was designed to identify EMs that can be reproducibly recorded and to assess individual variability in sponge EM production. Each sampling replicate of EXP3 was performed by filtering 10 L of water on three DVB disks simultaneously over the same individual sponge. A total of three independent capture replicates was obtained per sponge species (see S2 and S3). Samples of the sponge were taken in order to prepare crude extracts that served as analytical references for the identification of specialized metabolites.

### Untargeted Detection of Chemical Features by Tandem Mass Spectrometry

**Figure 3:**
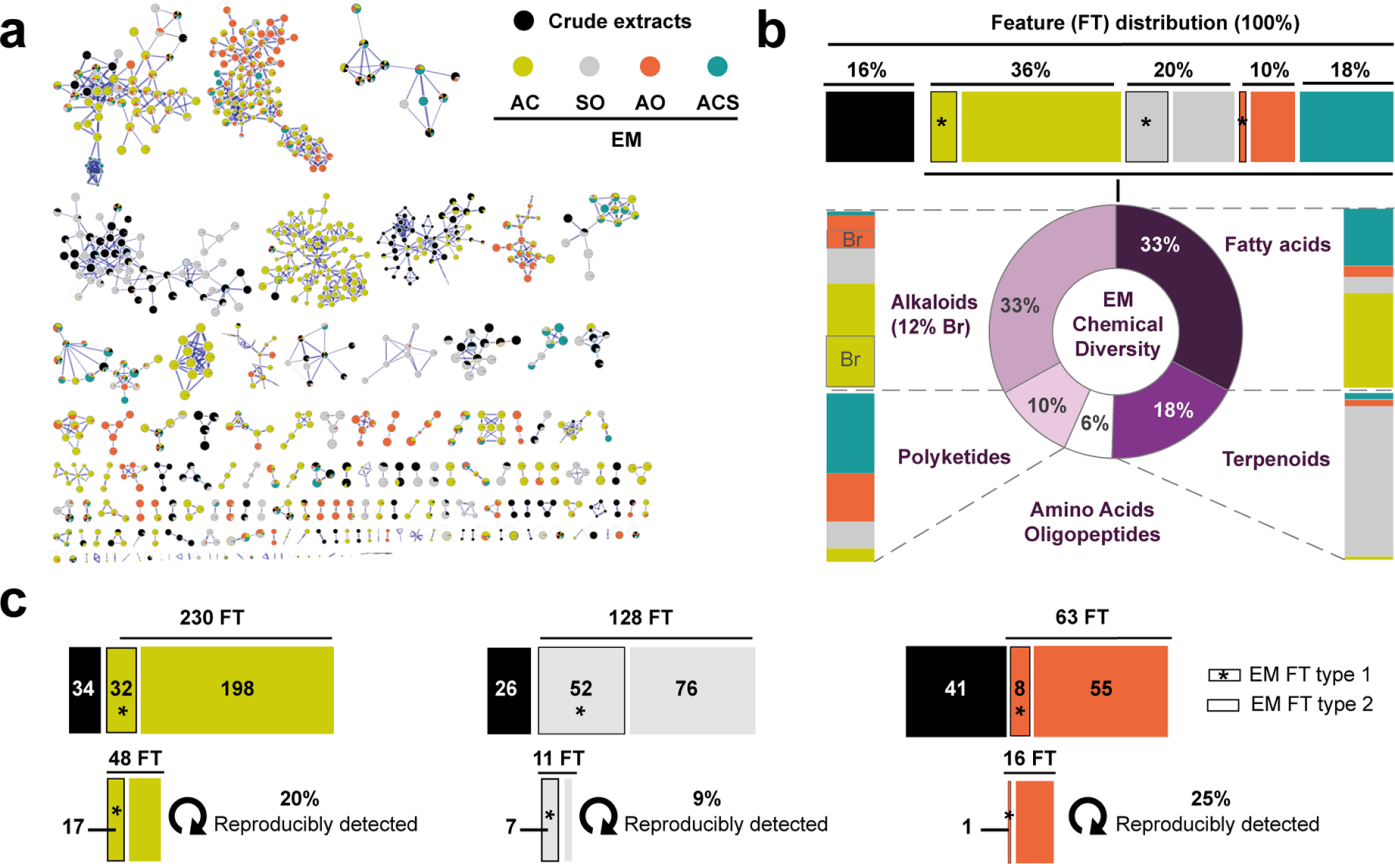
Chemical diversity of captured EMs. (a) Feature-based molecular network with ion identity containing 805 nodes organized in 137 spectral families (see also S4). The size of the node is proportional to the retention time. The pie chart represents relative intensities of features (FT) in each sample (ACS = average chemical seascape, AO = *Agelas oroides*, SO = *Spongia officinalis*, and AC = *Aplysina cavernicola*). (b) FT distribution and chemical diversity based on natural product pathway probabilities (>0.8) determined with CANOPUS.^42^ Sponge EM extracts contained three types of FT: identical to those from their crude extract (type 1), uniquely found as sponge-specific EMs (type 2), and clustering with features from ACS (type 3 in green). (c) Total number of FT for each sponge species and their distribution showing reproducibly captured EMs in sampling replicates (EXP2-3).

Back in the laboratory, metabolites adsorbed on SPE disks were eluted by using an automatic SPE instrument. Individual disk extracts were pooled per capture replicate, yielding a total of three replicates of the EM extracts for each series of experiments. All extracts were analyzed by ultra-high-performance liquid chromatography coupled to quadrupole time-of-flight tandem mass spectrometry detection with positive electrospray ionization. For comparison, all of the data were acquired during the same analytical sequence. The subsequent raw data were processed with MZmine 3^8^ to obtain a matrix characterized by a comparative listing of chemical features (MS^1^) with their corresponding ion intensities distinguished by their mass-to-charge ratio (*m*/*z*) and retention times. A matrix containing 2248 unique mass-retention time features with their ion intensities was subjected to GNPS together with the spectral MS^2^ data. Subsequently, a feature based molecular network^38^ (FBMN, Figure 3a) with ion identity^39^ was built to cluster together ion features (nodes) harboring similar MS^2^ spectra based on cosine scores (edge). This analysis was combined with a systemic interrogation of GNPS associated reference libraries. FBMN expands the interpretability of untargeted MS^2^ as it allows visualization of the spectral diversities and similarities within the studied data set, thus representing the chemical diversity of captured molecules.

### Characterization of EM Chemical Diversity and Variability Using FBMN

A total of 805 features were distributed in 137 spectral families (clusters; Figure 3a). With SIRIUS,^10^ we additionally focused on higher confidence assignment of molecular formulas in each targeted FBMN spectral family and looked into the distribution of predicted chemical class with CANOPUS.^36,42^ This led to a deeper molecular annotation of our data sets to describe the chemical diversity of collected molecules (Figure 3b). Sponge EM extracts contained three types of features: identical to those from their corresponding sponge extract (type 1), uniquely found as sponge-specific EMs (type 2), and clustering with features from ACS (in green, type 3). EM extracts from repeated seawater filtering around each species displayed distinct molecular compositions. In particular, type 1 features clearly suggested the presence of sponge-specialized metabolites as EMs. Type 2 features revealed the presence of EMs either structurally related to sponge-specialized metabolites or resulting from metabolic activities of other syntopic species (e.g., corals, bryozoans, and free-living micro-organisms) within the sponge community. Among all three species, *A. cavernicola* provided the highest number of detected features (230 features, Figure 3c). EMs obtained above *A. cavernicola* and *A. oroides* were found to be rich in alkaloids, including brominated ones, in addition to fatty acids and polyketide derivatives. Both sponge species are known to produce bromotyrosine spiroisoxazolines and bromopyrroles.^19,31^ Likewise, EMs collected around *S. officinalis*, known to produce diverse furanoterpenes and other terpenoids,^33^ were found to belong principally to the terpenoid structural class (Figure 3b). Overall, the accumulated captures (EXP2) recorded 52 EM features identical to those of *S. officinalis* specialized metabolites, 32 for *A. cavernicola*, and only 8 for *A. oroides* (Figure 3c). When focusing on reproducibly detected features (EXP2-3), these numbers decreased to 7 for *S. officinalis*, 17 for *A. cavernicola*, and only 1 for *A. oroides*. Finally, type 3 features accounted for 18% of all assigned signals, mainly in the fatty acid and polyketide structural classes. Such ACS features highlighted the presence of coralligenous EMs and other molecules that are not specifically related to the targeted sponge species. I-SMEL has thus demonstrated its capacity to capture a high chemical diversity around sponge species, with several specialized EMs defining the chemical fingerprint of each species’ surrounding seawater.

### Identification and Relative Proportion of Captured Sponge-Specialized EMs

To determine the structural identity and proportion of reproducibly captured specialized EMs for each sponge species, MS-based dereplication was performed using spectral data available either from previously identified compounds or in GNPS libraries, showcasing the usefulness of raw data sharing.^9^ When precise structural identification could not be achieved due to the lack of available reference raw data, we performed manual annotation of MS^2^ spectra corroborated with information disclosed in publications and with *in silico* prediction of molecular structures from MetFrag or CSI:FingerID embedded in SIRIUS.^10−12^ Metabolites identified using library match and *in silico* analyses are proposed with level 2b confidence as described previously.^43^ The proportion of each annotated EM was determined by measuring the area under the curve (AUC) of extracted ion chromatograms (EIC) in each extract from EXP2, using the crude as reference.

**Figure 4:**
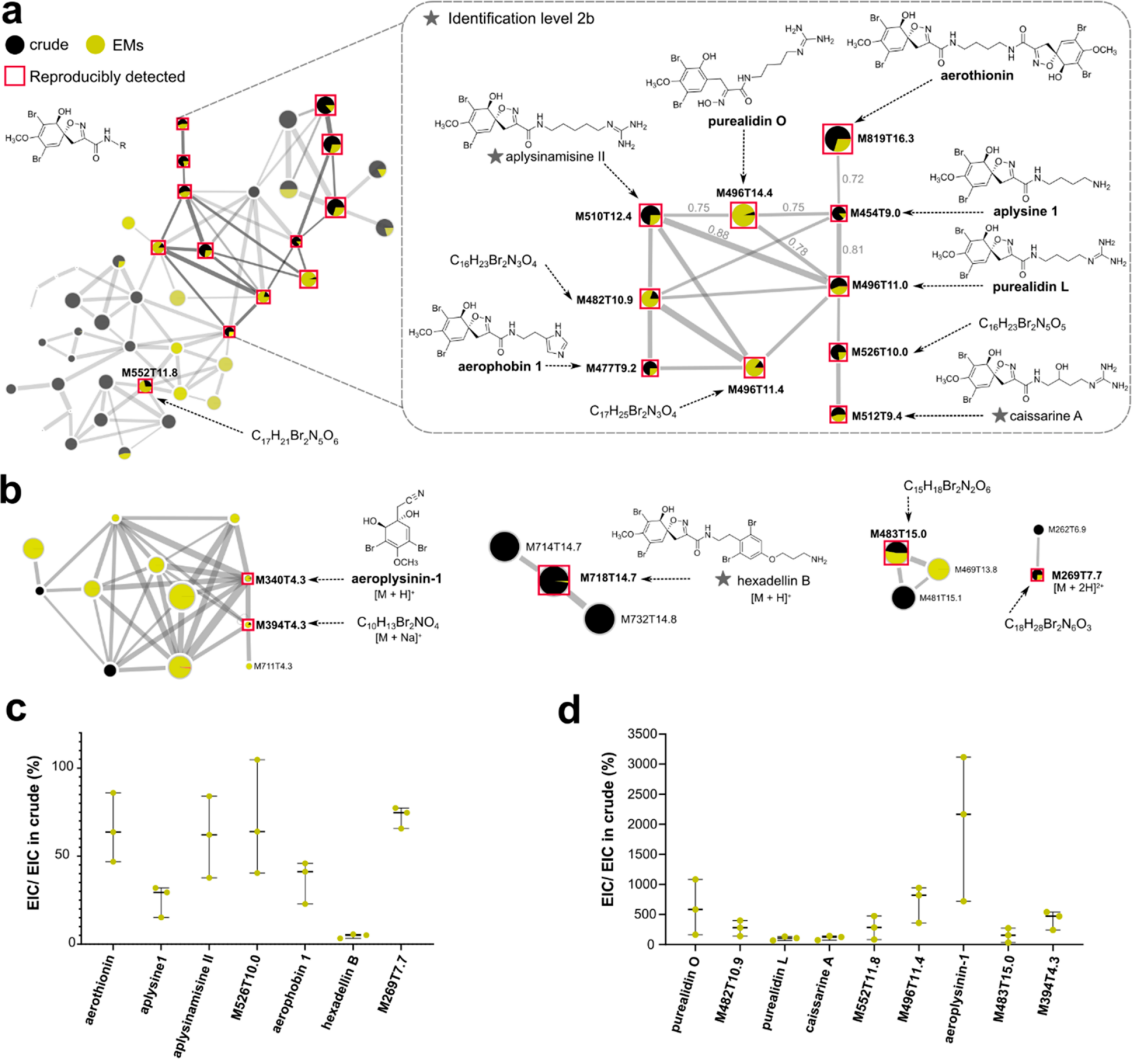
Spectral families and structural diversity of reproducibly enriched *A. cavernicola* specialized EMs. (a) Focus on the major bromo-spiroisoxazoline spectral family with the reproducibly detected features used for structural dereplication. Each feature is identified by its *m*/*z* (M) and retention time (T). The pie chart represents relative intensities of features in each sample. (b) Focus on four other spectral families, each containing one brominated alkaloid detected in both crude and EM extracts (confidence of identification level 2b for molecules associated with a star). (c and d) The relative concentration of each annotated EM was determined by measuring the area under the curve (AUC) of extracted ion chromatograms (EIC) in each extract and using the crude extract as reference (100%). (d) Metabolites more concentrated in EM extracts than in the crude extracts.

The sponge *A. cavernicola* was found to reproducibly release the highest number of specialized EMs. They corresponded to 16 brominated alkaloids (Figure 4), distributed in 5 spectral families. Six were previously described as EMs in aquarium experiments.^19^ In adequation with previous results, the most abundant bromo-spiroisoxazoline alkaloids characterizing the crude extract (Figure S5.1) were reproducibly detected as EMs, namely, aerothionin, purealidin L, aerophobin 1, aplysine 1, aplysinamisine II, and aeroplysinin-1. The nine other metabolites were either putatively identified by analyzing their MS^2^ spectra with regard to data available in the literature (Figure S6.1)^43^ or simply annotated with their molecular formula. Therefore, I-SMEL succeeded in concentrating a series of yet unknown bromotyrosine derivatives. Reproducibly detected specialized EMs belong to two groups based on their relative proportions: those with similar or lower concentration as in the crude extract (Figure 4c) and those whose concentration was significantly higher (Figure 4d). Among them, aeroplysinin-1, the most polar EM, was found to be present in the highest proportion. Such a difference could have been due to (1) different SPE extraction procedures between EM and crude extracts and (2) a higher solubility of aeroplysinin-1 favoring its enrichment from seawater.

**Figure 5:**
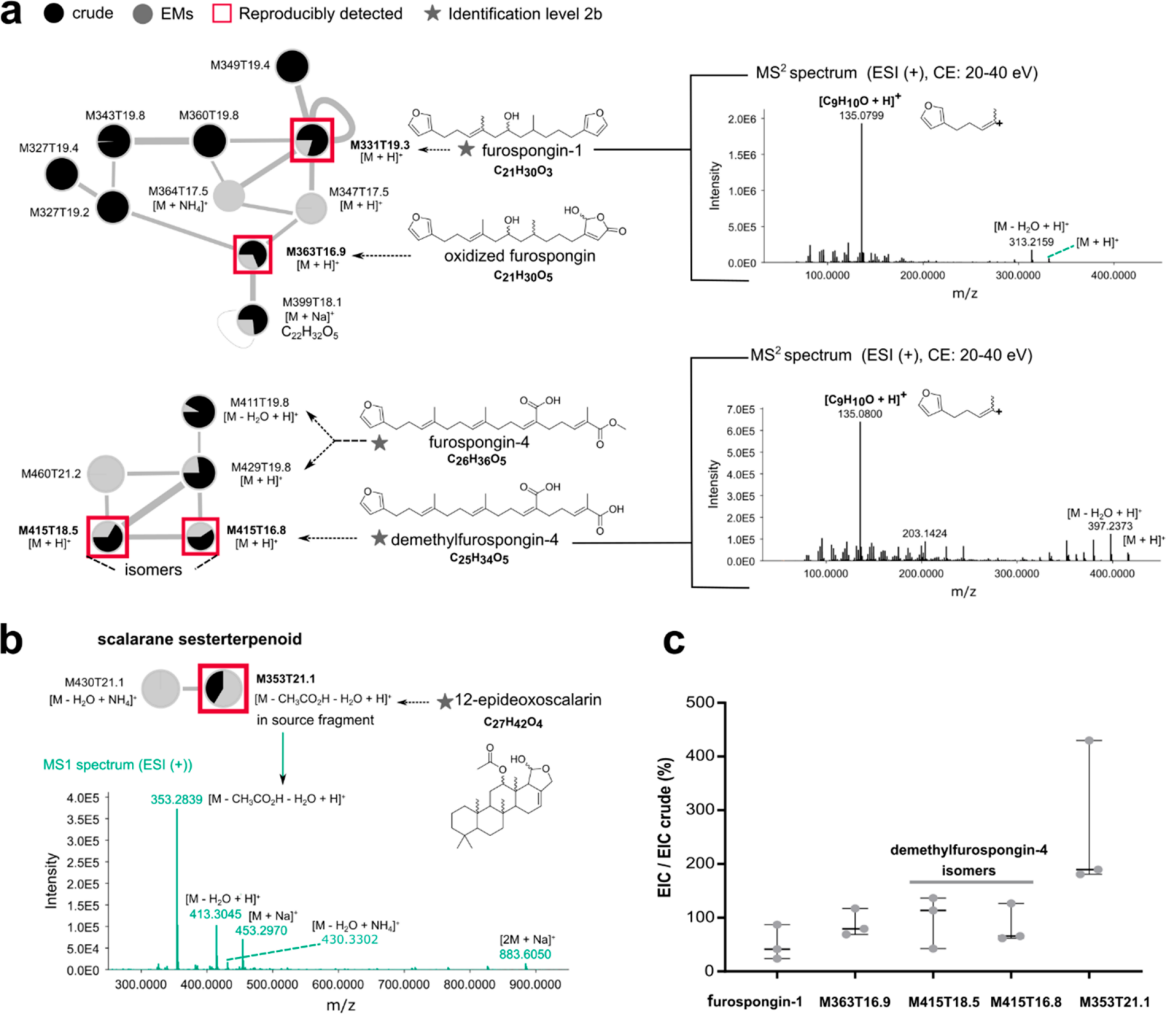
Spectral families of reproducibly released *Spongia officinalis* EMs. Each feature is identified by its *m*/*z* (M) and retention time (T). The pie chart represents relative intensities of features in each sample. (a) Focus on the furanoterpenoid spectral families with MS^2^ spectra of putative furospongin-1 and demethylfurospongin-4 showing a characteristic fragment at *m*/*z* 135.0800 corresponding to the furan fragment [C_9_H_10_O + H]^+^. Structural dereplication was performed in agreement with previously reported data^33,44^ (confidence level 2b). (b) M353T21.1 is an in-source fragment of the same molecule as M430T21.1, a scalarane sesterterpenoid. (c) The relative concentration of each annotated EM was determined by measuring the area under the curve (AUC) of extracted ion chromatograms (EIC) in each extract (EXP2) and using the crude extract as a reference (100%).

Around *Spongia officinalis* (SO), seven features representing seven molecules were reproducibly identical to those detected in the crude extract. Their MS^2^ spectra belonged to five distinct spectral families (Figure 5). The most representative ones encompass the highest number of MS^2^ spectra related to furanoterpenoids, as previously reported (Figure 5a).^33^ In this spectral family, furospongin-1, one of the most abundant specialized metabolites in *S. officinalis* (Figure S5.2), and an oxidized furospongin M363T16.9, were reproducibly identified as specialized EMs. Two isomers of dereplicated demethylfurospongin-4^33^ were also reproducibly detected in another spectral family encompassing furospongin-4, their methylated analogue. Another reproducibly released EM, M353T21.1, was found to be an in-source fragment ([C_27_H_42_O_4_ – CH_3_CO_2_H – H_2_O + H]^+^, *m*/*z* 353.2840) of the same molecule as M430T21.1 ([C_27_H_42_O_4_ – H_2_O + NH_4_]^+^, *m*/*z* 430.3317), annotated as 12-epideoxoscalarin, which was previously reported in *Spongia lamella* (Figure 5b, Figure S6.2).^44^ The last two metabolites were putative fatty acid derivatives (Table S6.2), and thus, they were not considered *stricto sensu* as specialized metabolites. The relative proportion of the oxidized furospongin M363T16.9 as well as both demethylfurospongin-4 isomers was found to be equivalent to those in the crude extracts (Figure 5c), whereas the relative concentration of M353T21.1 was on average twice as high in the EM extracts, emphasizing the enrichment capacity of I-SMEL.

**Figure 6:**
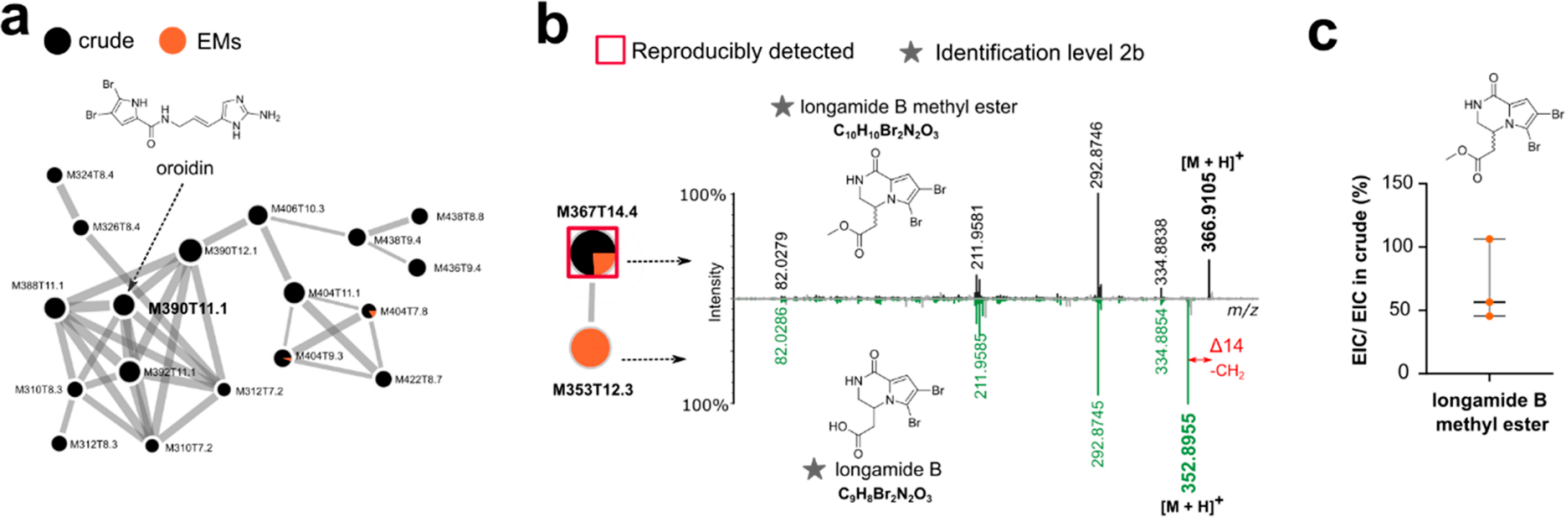
Spectral families representing the reproducibly enriched specialized EM from *Agelas oroides*. Each feature is identified by its *m*/*z* (M) and retention time (T). The pie chart represents the relative intensities of features in each sample. (a) Main spectral family related to AO and containing the oroidin MS^2^ spectrum. (b) Putative longamide B methyl ester, clustering with longamide B, is reproducibly detected as EM around the sponge (confidence level 2b). (c) The relative concentration of such EM is twice lower on average than in the crude extracts.

Among the bromopyrrole 2-amino-imidazole derivatives reported to be produced by *A. oroides* (AO), oroidin is by far the most abundant (Figure S5.3). In both the cumulative captures (EXP2) and individual replicates (EXP3), oroidin was not recovered as a specialized EM (Figure 6a). Only one brominated feature was repeatedly detected as EM (Figure 6b). Its molecular formula was calculated as [C_10_H_10_Br_2_N_2_O_3_ + H]^+^ (*m*/*z* 366.9105). Structural dereplication of its MS^2^ spectrum by manual annotation and using either SIRIUS or MetFrag^10,11^ led us to propose longamide B methyl ester (syn: hanishin methyl ester) as a putative structure (Figure S6.3). This compound was previously described in different *Agelas* species^31,45,46^ and clustered with the sporadically detected unique EM feature M353T12.3 ([C_9_H_8_Br_2_N_2_O_3_ + H]^+^, *m*/*z* 350.8974) assigned as longamide B.^45^ The relative concentration of longamide B methyl ester was found to be on average twice lower in the EM extracts than in the crude extracts.

## Discussion

Some 40 years ago, the first submersible apparatus was designed to capture metabolites around corals^23^ but was operated only in shallow water and with a relatively long sampling time, which together with the technical limitations of the time restrained the scope of its applications. Nevertheless, such a record illustrates clearly the persistant need for versatile *in situ* instruments capable of concentrating efficiently diluted metabolites in seawater. As opposed to previously developed *in situ* instruments, I-SMEL is hand-held and can thus travel with scientific divers. It can be deployed at various depths (here 15–20 m), under different configurations, allowing experiments to be perfomed in a time frame compatible with SCUBA diving. I-SMEL does not need to stay flat or be maintained on a flat surface to work. Collectively, these technical characteristics make I-SMEL stand out compared to previously developed *in situ* devices.^23,26,28^

The results presented herein showcase the ability of I-SMEL to recover structurally diverse metabolites including specialized EMs from the seawater surrounding a benthic ecosystem. We demonstrated that implementing I-SMEL followed by the described extraction process afforded samples significantly enriched in specialized EMs produced by sponges, such as brominated alkaloids and furanoterpenoid derivatives. Collectively, these EMs constitute the seawater chemical fingerprint of targeted sponge species, as also observed by ^1^H NMR analyses (Figure S7). The diversity and proportion of recovered specialized EMs were found to greatly differ between species but also between biological replicates of the same species, prefiguring an individual varability in EM production that would require a more thorough evaluation, as has been done for several other examples of sponge global metabolite production.^47,48^ Also, only a fraction of the known specialized metabolites characterizing a sponge crude extract were recovered as EMs. Some of them might have remained undetected due to (1) their presence in trace quantities below the detection threshold or (2) potential ionization suppression attributed to residual sea salts. A significant part of those specialized metabolites is also likely retained within the sponge body mass as a potential defense mechanism against predators or competitors.^49^

For the two most productive species studied here, *S. officinalis* and *A. cavernicola*, the most abundant specialized metabolites characterizing the crude extract were reproducibly recovered as EMs: furospongin-1 and demethylfurospongin-4 for *S. officinalis* and aerothionin and aerophobin-1 for *A. cavernicola*. This was not the case for *A. oroides*, considering that the most abundant specialized metabolite, oroidin, was not recovered as an EM. Instead, longamide B methyl ester, produced by different *Agelas* species, was reproducibly detected. EMs structurally deriving from well-known specialized metabolites were also sporadically detected around each of the three sponge species. Such results suggest that part of their released specialized metabolites may have been readily (bio)transformed before their capture. Possible (bio)transformation could produce more polar, water-soluble compounds, as observed with longamide B around *A. oroides* but also with oxidized furanoterpenes around *S. officinalis*. In line with such observations, aeroplysinin-1, a well-known bioactive bromotyrosine derivative,^50^ was proportionally more abundant in seawater extracts. To that extent, studying marine EMs helps revisit marine natural product chemistry, raising new questions regarding structural stability and water solubility and opening new prospects for the discovery of natural products. Even though this instrument has so far been tested only on keystone Mediterranean sponge species, I-SMEL could easily be deployed to explore the chemical diversity of EMs that might be released by other marine organisms (e.g., corals and algae).

The results gathered herein confirmed that I-SMEL can be used as a noninvasive/destructive technique facilitating the standardized sampling of seawater to efficiently capture, rapidly enrich, and thereafter monitor over time and space the diversity of EMs released by marine organisms. Using I-SMEL will enable the progressive mapping of chemical seascapes in various marine ecosystems and the qualitative and quantitative monitoring of EM production by specific marine organisms. The chemical knowledge gained from such future time-series studies could illuminate metabolic changes in the composition of the water column above benthic marine organisms. It may thus allow the highlighting of the chemical fluxes regulating organismal interactions (e.g., spawning, organism movements, and settlements) but also resolve new aspects of physiological responses, some of them induced by environmental stressors (e.g., pollutants and ocean warming).^51^ Moreover, stress biomarkers released in response to environmental disturbances could be directly captured with I-SMEL without manipulation or the destruction of marine organisms, thereby providing noninvasive means of early detection and monitoring.^14,52^ These applications represent game-changing advancements in the fields of marine and chemical ecology. I-SMEL could also be deployed to access and track *in situ* the distribution of dilute anthropogenic pollutants,^53^ opening new perspectives of investigations in the field of environmental chemistry. When combined with a thorough untargeted metabolomics workflow, the deployment of I-SMEL will assist in gaining knowledge of key marine ecosystems, including those harboring endangered species such as *Spongia officinalis*.^54^

In conclusion, geographical and seasonal investigations of enriched EMs from a given ecosystem become possible with I-SMEL. Results from such studies will provide crucial insights into the structuration of chemical mediation that underpins ecological interactions.^21^ Such results will also guide chemists in identifying the most productive periods during which appropriate seawater sampling above targeted marine organisms will provide higher quantities of extracts compatible with natural product discoveries. New generations of the easy-to-build I-SMEL will be of different sizes and autonomous and/or remotely operated and can be produced in order to adapt to different needs and logistical settings. With a collaborative network of marine researchers, these I-SMELs could be deployed simultaneously in different ecosystems, providing synchronized knowledge on chemical seascapes.

## Methods

### *In Situ* Solid Phase Adsorption Instrument

The main functioning items are contained in a 30-cm-diameter, 32-cm-high plexiglass cylinder (total volume 22 L). Most technical elements are in the upper part (Figure 1b,c), while the lower part forms a 10 L chamber holding three SPE supports (Figure 1a–c, S1). The upper part consists of an ERDEMIL peristaltic dosing pump coupled to a Maxon microreductor motor, powered by a 12 V, 250 mA rechargeable NiMH battery and controlled by a Pic16F electronic card. Such a system ensures repeatable and precise peristaltic pumping and can be operated regardless of the external pressure, thus without the diving depth affecting the volume of pumped seawater. All of these elements are contained in custom-built waterproof stainless steel housings. The microcontrollers on the electronic card allow the pumping parameters (i.e. pumping duration, volume of pumped seawater, and therefore the flow rate) to be set. The parameters are set before the dive, and the exact delivered volume can be checked at the beginning of each dive by connecting a flexible tank to the pump outlet (Figure 1a, S1). A defined volume of seawater can be pumped repeatedly as many times as needed. A simple waterproof magnetic push button triggers pumping (short press) whatever the position of the device. The pump is connected to three suction ports that can be opened or closed by small valves operated underwater. Each suction port is equipped with a filter holder in which a stainless steel grid with a 2 mm mesh retains the coarsest particles, with the SPE disks being right behind the grid. With the exception of the stainless steel grids and the plexiglass chamber, all of the components in contact with the pumped seawater are made of PTFE.

### *In Situ* Adsorption and Concentration of Sponge Exometabolites (EMs)

*In situ* experiments were performed by SCUBA diving in the Marseille area in Calanques National Park. This study targeted the coralligenous community distributed at the entrance of underwater caves. During each dive, the instrument was placed above an assemblage of species that are, under such conditions, dominated by marine invertebrates, mostly sponges, but also corals and bryozoans. DVB disks (47 mm Atlantic Disk Biotage) were mounted on the SPE supports after being individually conditioned with 10 mL of ethyl acetate (EtOAc), followed by 10 mL of methanol (MeOH) and ending with 10 mL of distilled water (H_2_O). The first experiment aimed at obtaining the average chemical seascape of this marine ecosystem by performing a 10 min capture in total, with I-SMEL being moved at least five times in close contact with random parts of the ecosystem (EXP1). A second series of 10 min captures (10 L of pumped seawater) was performed by targeting the sponges *Aplysina cavernicola*, *Spongia officinalis*, and *Agelas oroides* in order to obtain three average seascapes respectively enriched in the exometabolites of these three species (EXP2). To maximize our chance to trace the impact of each target species on the chemical seascape, 2 L of seawater (2 min duration) was repeatedly sampled above 5 parts of the ecosystem dominated by each species. Each capture replicate was performed with all valves opened on the three DVB disks to yield one enriched sponge EM extract. A total of three extract replicates were produced for each sponge species. The last type of experiment (EXP3) was performed to get biological replicates and therefore preliminarily assess the individual variability by realizing three replicate captures of 10 min each on different specimens of sponge species (sampling Scheme S2).

### Extraction of *In Situ* Adsorbed and Enriched EMs

The extraction of metabolites adsorbed on the DVB disks was processed on an automatic solid phase extraction instrument (Dionex Autotrace 280, Thermo Scientific). The disks were washed with 30 mL of deionized H_2_O as the desalting step. EM extraction was performed by eluting the DVB disks with 10 mL of MeOH, followed by 10 mL of MeOH/EtOAc (50:50 v/v) and ending with 10 mL of EtOAc. A 30 mL EM extract was obtained, evaporated to dryness (SpeedVac, Savant SPD111 V), rediluted in 1 mL of MeOH MS-grade, and kept for at least 2 h at −20 °C to precipitate the residual sea salts. After centrifugation (5 min at −4 °C, 14K rpm), the supernatants were filtered on PTFE luer-lock filters (0.22 μm, cat. no. 26142, Restek). Extracts from the three DVB disks used per capture experiment were pooled to produce one EM extract replicate, thus yielding three ACS extracts (EXP1) and three EM extracts per sponge species and type of experiment (total 18 EM extracts EXP2-3).

### Preparation of Sponge Crude Extracts

All samples were collected in agreement with French national regulations and the Nagoya Protocol, processing with a French national declaration under receipt no. TREL2022990S I 392 and the internationally recognized certificate of compliance (IRCC) number ABSCH-IRCC-FR-253848-1 available from the Access and Benefit-Sharing Clearing-House (https://absch.cbd.int/about/). Soon after collection, all sponges were transported in sealed jars of seawater without exposure to air. In the laboratory, sponges were flash frozen in liquid nitrogen, stored at −80 °C, and then freeze-dried for at least 48 h. The dried sponge was reduced to a thin powder with a knife mill for domestic use. Approximately 1 g of each sponge powder was mixed with 10 mL of MeOH LC-grade and sonicated for 30 min (ultrasonic cleaner TUC-70). The mixture was filtered under vacuum, and the remaining sponge powder was rinsed with 10 mL of MeOH. Crude salted extracts adsorbed on 100 mg of C-18 powder (Polygoprep 60-50, Macherey-Nagel) were further processed on Strata C18-E SPE cartridges to remove residual sea salts. Extracts were washed with 18 mL of LC-MS-grade water and then eluted with 12 mL of MS-grade MeOH, thereby leading to the production of desalted sponge crude extracts. These extracts were used as analytical references.

### UHPLC-UV HR-MS Acquisition

Sponge crude extracts were prepared at 0.2 mg·mL^–1^ in MS-grade MeOH and filtered on PTFE luer-lock filters (0.22 μm, catalog no. 26142, Restek) prior to injection. Chromatographic separations were achieved on a Luna Omega Polar C18 UHPLC column (Phenomenex), maintained at 42 °C, with an elution gradient composed of (A) water and (B) MeCN both with 0.1% formic acid under the following conditions: from 10% (B) during 2 min to 29.5% (B) at 13.3 min and then to 95% (B) at 17 min and during 4.5 min (flow rate 0.45 mL·min^–1^, injection volume 2 μL). Mass spectrometry detection parameters (ESI-Q-ToF, Bruker Impact II) in ESI positive mode were set as follows: nebulizer gas N_2_ at 3.5 bar; dry gas at 12 L·min^–1^; capillary temperature at 200 °C; and voltage at 4500 V. MS/MS acquisition mode was set with a scan rate of 8 Hz (full scan from 50 to 1200 *m*/*z*) and a mixed collision energy of 20–40 eV (50% time at each collision energy, stepping mode). A sodium formate/acetate solution forming clusters in the studied mass range was used as the calibrant and automatically injected before each sample for internal mass calibration, ensuring a precision of *m*/*z* lower than 2 ppm in the mass range. Extracts were randomly injected to integrate any memory effect on the column and time-dependent MS drift. Pooled samples, injected every six samples from the beginning to the end of the series, were used for further ion filtering. See S6 for UHPLC-MS data processing with MZmine 3.2.8.^55^

### Molecular Networking and Analysis of Chemical Diversity

The feature quantification table and the corresponding list of MS^2^ spectra linked to the 2248 MS1 features (mgf file format) were exported from MZmine^55^ for feature based molecular networking on the GNPS platform (https://gnps.ucsd.edu).^9,38^ The precursor ion mass tolerance was set to 0.02 Da, and the MS/MS fragment ion tolerance was set to 0.02 Da. A molecular network was then created where edges were filtered to have a cosine score above 0.7 and more than 10 matched peaks in the MS^2^ spectra. Furthermore, edges between two nodes were kept in the network only if each of the nodes appeared in each other’s respective top eight most similar nodes. Finally, the maximal size of a molecular family was set at 100, and the lowest scoring edges were removed from molecular families until the molecular family size was below this threshold. The acquired MS^2^ spectra in the network were then searched against GNPS spectral libraries.^9^ The resulting network was visualized and interpreted using Cytoscape 3.8.2.^56^ Spectral families were individually processed using *in silico* tools in SIRIUS 5.6.^10^ Higher confidence assignment of molecular formulas for features within each spectral families and structural predictions were made using embedded as well as in-house-built databases with the simplified molecular input line-entry system [SMILES]. CANOPUS was then used for systematic annotation of the compound class of each selected structural prediction. Chemical distribution and classification of all clustered features were performed using outcomes from NPclassifier and using anatural product pathway probabilities of >0.8.^36,42^

### Proportion of Sponge-Specialized EMs

The relative proportion of sponge-specialized metabolites detected as EMs from EXP2 was determined by comparing the area under the curve (AUC) of their extracted ion chromatograms (EIC). The corresponding AUC measured in the sponge crude extract was used as reference (100%). Both EIC and their measured AUC were obtained using Compass DataAnalysis software (Bruker version 5.0). Results were interpreted by taking into account the concentration of sponge crude extracts (0.2 mg in 1 mL) and the volume used to reconstitute seawater EMs extracts (1.5 mL of concentrated extract from three DVB disks and 10 L of filtered seawater).

### Safety Statement

No unexpected or unusually high safety hazards were encountered.

## Data Availability

Raw MS^2^ data (*.mzML) are freely available from the UCSD Center for Computational Mass Spectrometry database with the MassIVE identifier MSV000091465. The molecular networking job can be publicly accessed at https://gnps.ucsd.edu/ProteoSAFe/status.jsp?task=cff712297d024e52a2c03bac310af869. The raw (*mzML) MS data, .mol files of all represented structures, Excel files used to determine the distribution of molecular features, the cytoscape file, Excel files pertaining to data presented in each figure, and ^1^H NMR data are available on ZENODO at https://doi.org/10.5281/zenodo.7820941.

## Supporting Information Available

Further details on the I-SMEL instrument; scheme of the sampling protocol; extracts codes and masses; complementary information to Figure 3; base peak chromatograms of representative sponge crude extracts; MS data processing as well as annotated MS^2^ spectra and corresponding data of (putatively) identified EMs; and ^1^H NMR spectra of sponge EM extracts (PDF)
Transparent Peer Review report available (PDF)
