# Supplementary material for: In Situ Capture and Real-Time Enrichment of Marine Chemical Diversity
Source: ACS Cent Sci. 2023 Nov 8;9(11):2084–95. doi: 10.1021/acscentsci.3c00661 (PMC10683479; doi:10.1021/acscentsci.3c00661)
Supplement: Supplementary file 1 — oc3c00661_si_001.pdf [file oc3c00661_si_001.pdf]

In situ Capture and Real Time Enrichment of Marine Chemical Diversity

Morgane Mauduit[#], Marie Derrien[#], Marie Grenier[#], Stéphane Greff, Sacha Molinari, Pierre Chevaldonné, Charlotte Simmler[¤,*], Thierry Pérez[¤]

[1] IMBE, UMR CNRS 7263, IRD 237, Aix Marseille Université, Avignon Université, Endoume Marine Station, Chemin de la batterie des lions, 13007 Marseille, France

[#] co-first authors

[¤] co-last authors

* corresponding author

Correspondence to: charlotte.simmler@imbe.fr; ORCID ID: 0000-0002-6923-2630.

Supporting Information

All the raw MS^2 data (*mzML) are freely available at the UCSD Center for Computational Mass Spectrometry database with the MassIVE identifier MSV000091465. MS and NMR data presented in this manuscript are also available on ZENODO at https://doi.org/10.5281/zenodo.7820941.

Contents

S1. Further technical details on I-SMEL **S3**

S1.1. Detailed pictures of the different parts **S3**

S1.2. Description of the SPE supports **S4**

S1.3. Change of SPE supports during a SCUBA dive **S5**

S2. *In situ* sampling protocol (EXP1-3) **S6**

S3. EM extracts from EXP1-3: codes and extraction yields **S8**

S4. Complement to Figure 3: Marine Chemodiversity through the analysis of FBMN **S10**

S5. Annotated Base Peak Chromatograms of sponge crude extracts **S11**

Figure S5.1. *Aplysina cavernicola* **S11**

Figure S5.2. *Spongia officinalis* **S11**

Figure S5.3. *Agelas oroides* **S11**

S6. MS data processing and annotated MS² spectra of reproducibly detected EMs **S12**

S6.1. *Aplysina cavernicola* **S13**

S6.2. *Spongia officinalis* **S19**

S6.3. *Agelas oroides* **S25**

S7. ^{1}H NMR spectra of sponge EM extracts (EXP2) **S25**

S7.1. *Aplysina cavernicola* **S26**

S7.2. *Spongia officinalis* **S28**

S7.3. *Agelas oroides* **S28**

S7.4. Stacked ^{1}H NMR spectra of all EM extracts (EXP2) **S29**

S1. Further technical details on I-SMEL

S1.1. Detailed pictures of the different parts

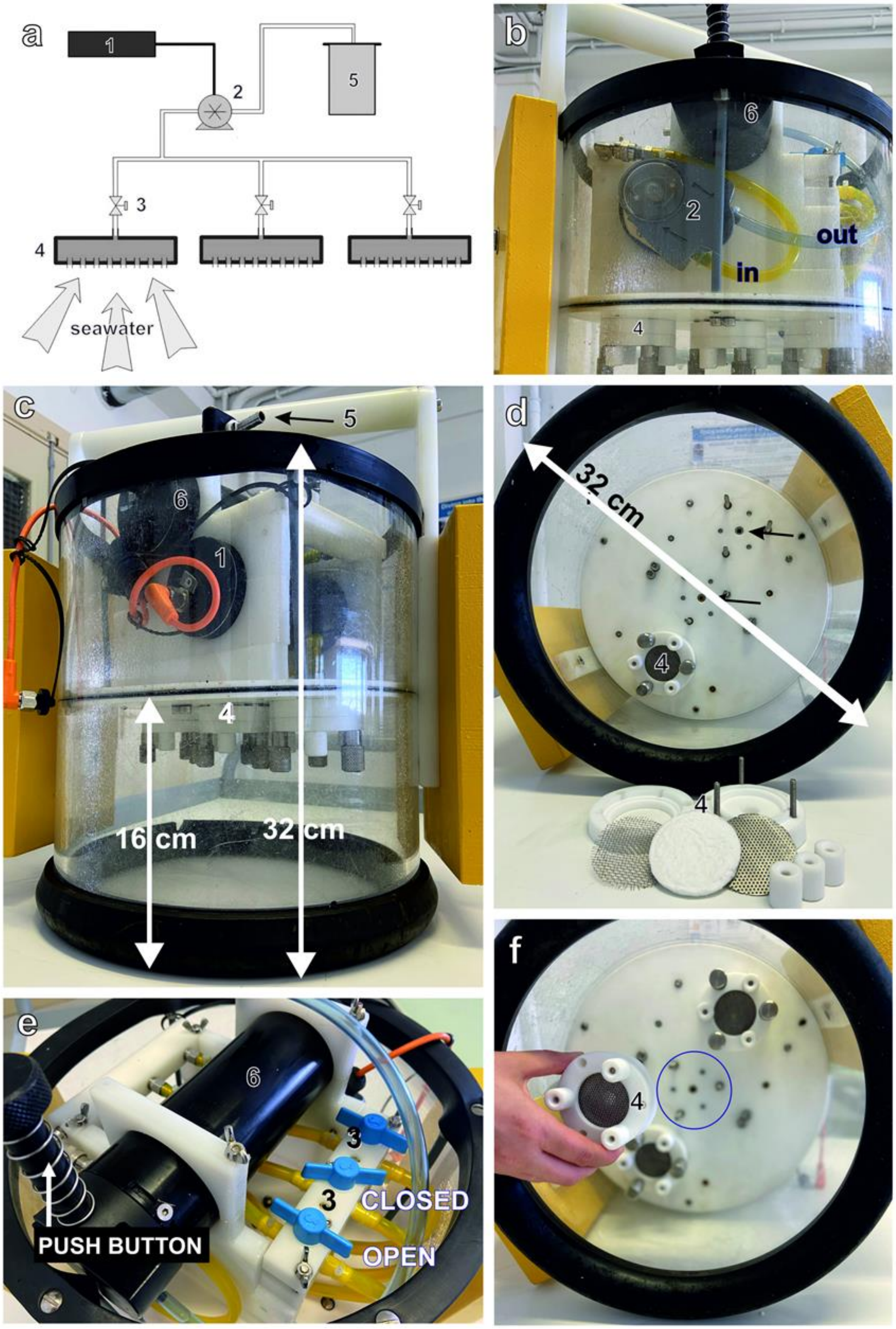

(a) Schematic view of the main items within I-SMEL: identification of the instrument items: **1** electronic controller of the pump, **2** peristaltic pump, **3** valves, **4** SPE supports, **5** pump outlet where a flexible container can be adapted, **6** the battery, (b) zoom in the upper part of I-SMEL containing the peristaltic pump, its controller and the battery in stainless steel enclosures, (c) closer overview on the entire instrument divided in two parts: the upper one with all the electronics and the pump outlet, and the lower one, where the SPE supports are tightly screwed and turned toward the bottom, thus toward the organisms to sample, (c-f) view inside the chamber, where the SPE supports have to be fixed one by one, (e) upside view of I-SMEL with the battery, the waterproof magnetic push button activating the pump and the three valves that can be maintained closed or opened allowing the seawater to go through a selected SPE disk.

Peristaltic dosing pump: ERDEMIL™ coupled to a Maxon™ microreductor motor
Battery: 12 V, 250 mA rechargeable NiMH battery
Controller: Pic16F electronic card
Connecting tubing: flexible PTFE tubing 8/11 mm diameter.
I-SMEL chamber is delimited by plexiglass. All the other parts are made of PTFE.

Notes The instrument is made of six primary components as shown here and in figure 1 of the manuscript. The dimensions of the capture chamber can be adjusted according to the research objectives or the target organism. Similarly, the peristaltic pump's specificity (*e.g.* flow rate) can be tailored to match the intended purpose. The sizes of the two stainless steel enclosures that house respectively the battery and electronic controller are also contingent upon the desired level of autonomy. These two enclosures, along with the electronic controller, were constructed in-house.

S1.2. Description of the SPE supports

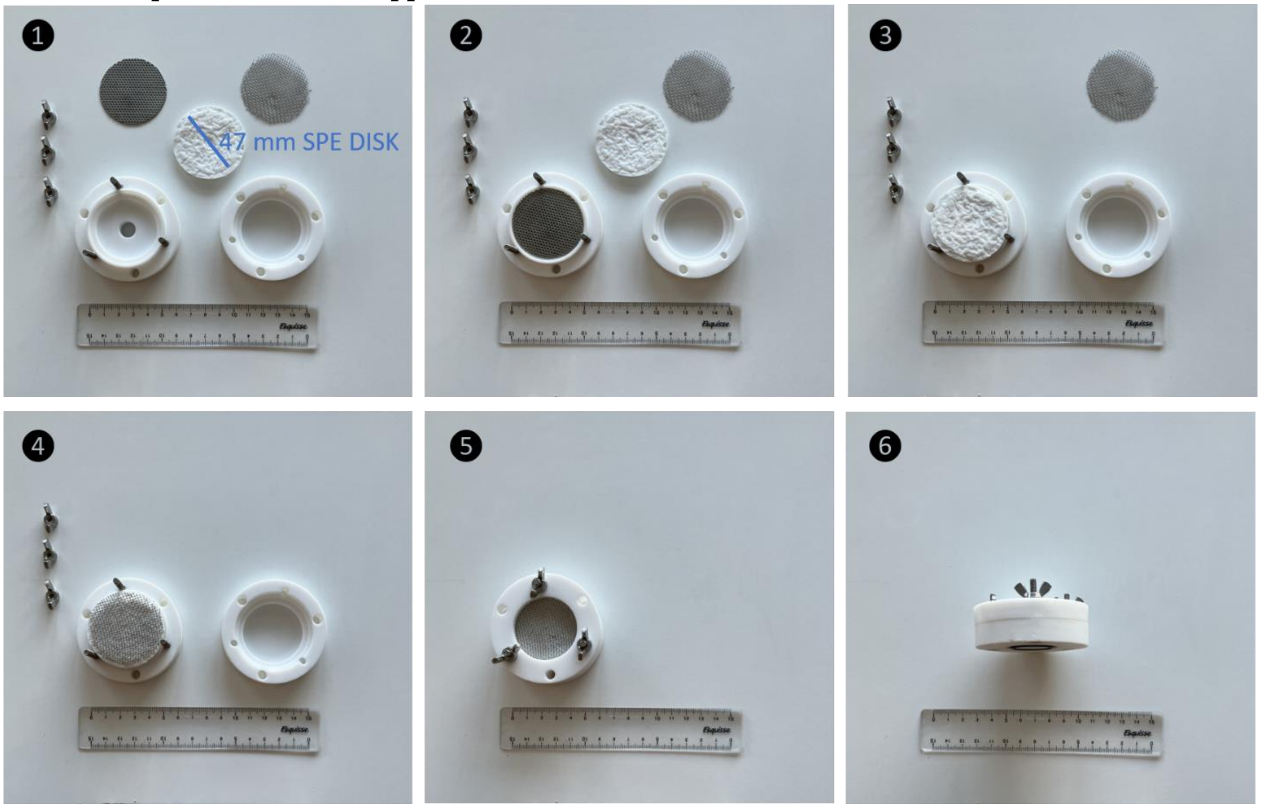

Figure S1.2. Illustration of how the SPE disks (47 mm diameter) are installed inside the SPE supports, which are made of 4 different parts to assemble. Two PTFE cases and two stainless steel grids of different mesh. The grid on top of the SPE disk has a 2 mm mesh which retains the coarsest particles (4). The preconditioned SPE disk is placed inside the holder prior to the dive. All four parts enclosing the disk are maintained tightly together by outside screws (5, 6).

S1.3. Change of SPE supports during a SCUBA dive

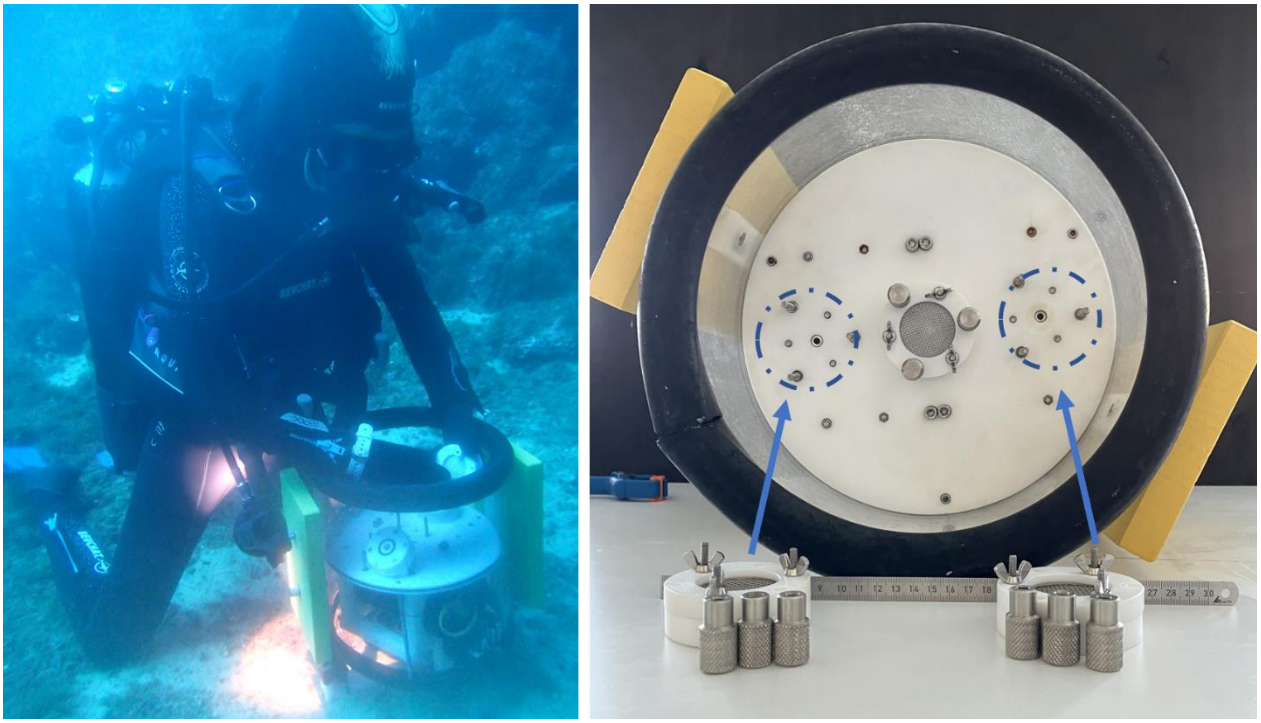

Figure S1.3. SCUBA diver changing the SPE supports prefilled with new SPE disks. The photo on the right shows the large stainless steel screws used to fix the SPE support inside I-SMEL. The diver has to remove then replace these screws to change the SPE holders.

S2. *In situ* sampling protocol (EXP1-3)

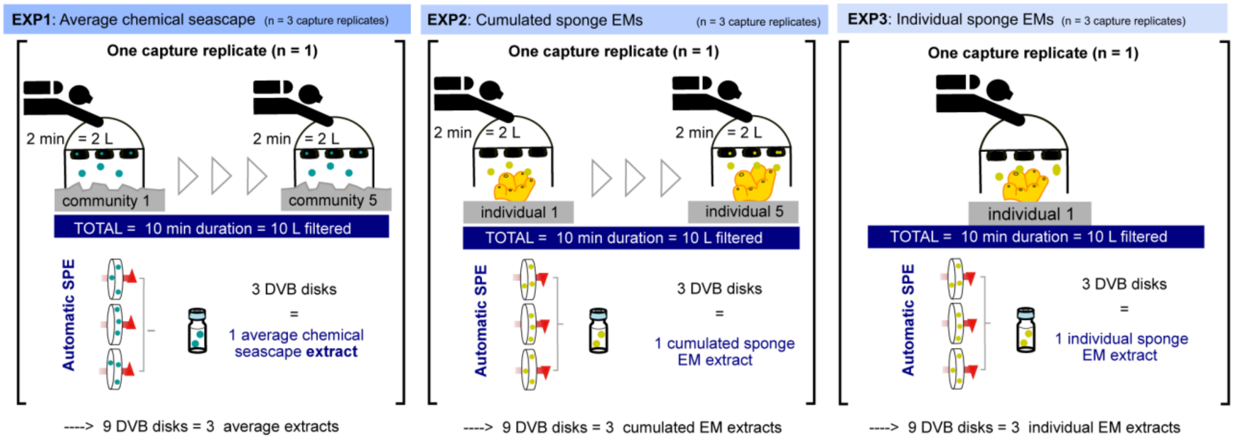

Figure S2.1. Sampling protocol used in this study for EXP1 to EXP3.

For EXP1 and 2: one replicate experiment corresponds to the filtration of a total of 2 L of seawater repeated above either 5 different community (EXP1) or 5 different sponge specimens (EXP2). During the sampling all three valves are opened. Therefore, for one replicate a total of 10 L of seawater is passed through 3 SPE disks simultaneously.

The three disks are then eluted individually then pooled to provide an average extract of marine chemical seascape (n = 1, EXP1) or an average extract of cumulated sponge EMs (n = 1, EXP2). Both EXP1 and 2 were replicated three times, resulting in 3 replicate extracts for each EXP.

- 1 replicate experiment = one average extract from 3 SPE disks simultaneously from **5 different substrates**. *In situ* sampling duration 5 x 2 min = sampling volume 10 L (5 x 2 L).

For EXP3: one replicate experiment corresponds to the filtration of 10 L of seawater above one sponge individual, on three SPE disks simultaneously. After individual elution of these disks, the solvents are combined to obtain one single extract enriched in individual sponge EMs. EXP3 was replicated three times to obtain 3 individual EM extracts/sponge species.

- 1 replicate experiment = one average extract from 3 SPE disks simultaneously from **1 single sponge individual**. *In situ* sampling duration 1 x 10 min = sampling volume 10 L.

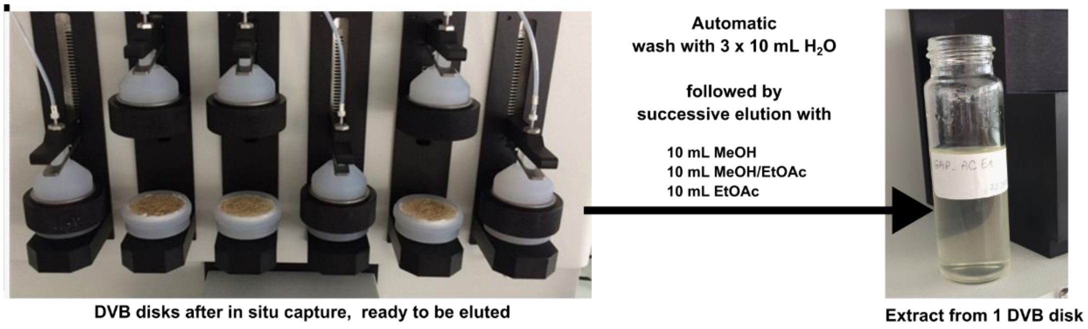

Figure S2.2. depicts three SPE disks from *in situ* capture ready to be eluted. Each elution provides a 30 mL extract/disk. These extracts are then pooled and dried to constitute one replicate sample, which is diluted in 1.5 mL MeOH MS-grade prior to LC-MS analysis.

S2.3. Notes on the rationale behind the choice of SPE DVB disks

DVB polymeric matrix: Among the different polymeric solid phases, DVB is the most used to enrich water-soluble natural products (Berlinck *et al*. 2021)[1] because it is able to retain structurally diverse dissolved metabolites of wide polarity range. As such, DVB solid phase (*e.g.* Bond-Elut-PPL) is used to capture Dissolved Organic Matter (DOM) from filtered seawater (Dittmar *et al.* 2008, Petras *et al.* 2017)[2,3]. DVB resins have also been previously deployed in device aiming at passively sampling microalgae toxins *in situ* (SPATT Solid Phase Adsorption Toxin Tracking [Roué *et al*. 2018])[4]. More recently, DVB resin (HP-20) lead to the isolation of metabolite directly enriched in the ecosystem (Bogdanov *et al.* 2023)[5]. Finally, previous work focusing on collecting EMs released by sponge in aquarium used DVB polymeric SPE (Vlachou *et al.* 2018)[6]. For all those reasons, we chose DVB as a polymeric phase.

The disks were preferred compared to the cartridges as they offer a larger surface of exchange, allowing faster flow rate to be applied, compatible with shorter sampling time. Also, in general, SPE disks are more appropriate when the water to be filtered is charged with different types of particulates. The disks were chosen instead of resins as they are, for now, easier to use and to change underwater and more importantly, compatible with a standardized SPE elution/extraction in the laboratory using automated SPE instruments.

As I-SMEL encloses three distinct SPE supports, it would be possible to perform *in situ* enrichment of exometabolites using different, yet complementary, SPE matrices (*e.g.* HLB and DVB).

S3. EM extracts from EXP1-3: codes and extraction yields

Table S3.1. Mass of eluted EM extracts from EXP1 and 2

EXP	Sample	CAPT	Replicate code/ DVB disk	Qty (mg)	Mean qty (mg)	Stand. Dev.
**1**	ACS	1	EXP1_ACS_CAPT1_R1	14.5	25.1	10.0
			EXP1_ACS_CAPT1_R2	34.4		
			EXP1_ACS_CAPT1_R3	26.3		
		2	EXP1_ACS_CAPT2_R1	86	45.4	35.4
			EXP1_ACS_CAPT2_R2	29.8		
			EXP1_ACS_CAPT2_R3	20.5		
		3	EXP1_ACS_CAPT3_R1	21.4	21.0	6.3
			EXP1_ACS_CAPT3_R2	27.1		
			EXP1_ACS_CAPT3_R3	14.5		
**2**	AC*	1	EXP2_AC_CAPT1_R1	16.7	16.5	1.3
			EXP2_AC_CAPT1_R2	15.1		
			EXP2_AC_CAPT1_R3	17.6		
		2	EXP2_AC_CAPT2_R1	3.5	17.2	19.3
			EXP2_AC_CAPT2_R2	8.7		
			EXP2_AC_CAPT2_R3	39.3		
	SO	1	EXP2_SO_CAPT1_R1	28.5	22.6	17.6
			EXP2_SO_CAPT1_R2	36.4		
			EXP2_SO_CAPT1_R3	2.8		
		2	EXP2_SO_CAPT2_R1	1.6	15.6	10.0
			EXP2_SO_CAPT2_R2	31.1		
			EXP2_SO_CAPT2_R3	12.1		
		3	EXP2_SO_CAPT3_R1	12.9	21.8	15.4
			EXP2_SO_CAPT3_R2	22.1		
			EXP2_SO_CAPT3_R3	14		
	AO	1	EXP2_AO_CAPT1_R1	29.6	19.6	11.9
			EXP2_AO_CAPT1_R2	22.8		
			EXP2_AO_CAPT1_R3	6.4		
		2	EXP2_AO_CAPT2_R1	16	11.2	5.2
			EXP2_AO_CAPT2_R2	11.9		
			EXP2_AO_CAPT2_R3	5.7		
		3	EXP2_AO_CAPT3_R1	32.2	23.4	11.5
			EXP2_AO_CAPT3_R2	27.7		
			EXP2_AO_CAPT3_R3	10.4		

* We had an experimental mishap with the third replicate and did not measure the extraction yields.

EXP: type of Experiment; CAPT: *in situ* capture replicate, n = 3, R: technical Replicate of DVB disk. Each capture was performed on the three DVB disks simultaneously. ACS = Average Chemical Seascape, SO = *Spongia officinalis*, AC = *Aplysina cavernicola*, AO = *Agelas oroides*.

Table S3.2. Mass of eluted EM extracts from EXP3

EXP	Community	CAPT	Replicate code/ DVB disk	Qty (mg)	Mean qty (mg)	Stand. Dev
**3**	AC	4	EXP3_AC_CAPT4_R1	25.2	27.4	9.2
			EXP3_AC_CAPT4_R2	19.5		
			EXP3_AC_CAPT4_R3	37.5		
		5	EXP3_AC_CAPT5_R1	20.4	30.1	11.8
			EXP3_AC_CAPT5_R2	26.6		
			EXP3_AC_CAPT5_R3	43.3		
		6	EXP3_AC_CAPT6_R1	17	18.0	12.2
			EXP3_AC_CAPT6_R2	6.3		
			EXP3_AC_CAPT6_R3	30.7		
	SO	4	EXP3_SO_CAPT4_R1	12.4	26.8	21.8
			EXP3_SO_CAPT4_R2	17.1		
			EXP3_SO_CAPT4_R3	52.3		
		5	EXP3_SO_CAPT5_R1	33	26.4	8.0
			EXP3_SO_CAPT5_R2	28.6		
			EXP3_SO_CAPT5_R3	17.5		
		6	EXP3_SO_CAPT6_R1	21.7	27.0	11.5
			EXP3_SO_CAPT6_R2	40.2		
			EXP3_SO_CAPT6_R3	19.1		
	AO	4	EXP3_AO_CAPT4_R1	43	34.1	15.5
			EXP3_AO_CAPT4_R2	43.2		
			EXP3_AO_CAPT4_R3	16.2		
		5	EXP3_AO_CAPT5_R1	51.9	38.5	12.0
			EXP3_AO_CAPT5_R2	28.7		
			EXP3_AO_CAPT5_R3	34.9		
		6	EXP3_AO_CAPT6_R1	52.3	31.4	22.3
			EXP3_AO_CAPT6_R2	33.9		
			EXP3_AO_CAPT6_R3	7.9		

EXP: type of Experiment; CAPT: *in situ* capture replicate, n = 3, R: technical Replicate of DVB disk. Each capture was performed on the three DVB disks simultaneously. ACS = Average Chemical Seascape, SO = *Spongia officinalis*, AC = *Aplysina cavernicola*, AO = *Agelas oroides*.

S4. Complement to Figure 3: Marine Chemodiversity through the analysis of FBMN

Table S4.1. Global FBMN node distribution

Total features (FT) = Total nodes	2248
Total single nodes, single loop	1376
Total single nodes included in spectral families	80
Total nodes distributed in 137 spectral families	872
Total nodes corresponding to technical contaminants	67
Total nodes in spectral families without contaminants	805

Table S4.2. Distribution[a] of features (FT) or nodes in the MN between identified groups

Species	Groupe (FT type)	Qty of Nodes	% / species	% of classified FT[b]
AC	crude only	34	13	5
	crude & EM (1)	32	12	**5**
	EM only (2)	198	75	**31**
Unique to AC		264	100	41
SO	crude only	26	17	4
	crude & EM (1)	52	34	**8**
	EM only (2)	76	49	**12**
Unique to SO		154	100	24
AO	crude only	41	39	6
	crude & EM (1)	8	8	**1.0**
	EM only (2)	55	53	**9**
Unique to AO		104	100	16
Marine ACS Metabolites (3)		116		**18**
TOTAL		643		100

[a]For all EXP1 to 3, [b]Values used in figure 3b.

A total of 764 FT was attributed to different sample groups. Out of them, 119 features were found to be detected in two or three sponge EMs, and did not cluster with any crude extracts or ACS samples. These features were not included in the representation of feature distribution as they were not specific to a given sponge community. See Excel spreadsheet 103 exomet_fig3R1vf_MN on ZENODO at https://doi.org/10.5281/zenodo.7820941.

Table S4.3. Evaluation of molecular diversity within the classified features

	Number of features per sample type				
NP pathway	**ACS**	**SO**	**AC**	**AO**	**TOTAL**
**alkaloids**	2	21	60	19	102
**amino acids-oligopeptides**	1	1	17	0	19
**polyketides**	15	5	3	9	32
**terpenoids**	2	50	1	2	55
**fatty acids**	32	10	53	6	101
**TOTAL NP > 0.8**	52	87	134	36	309

A total of 744 features were annotated using SIRIUS, 488 of them had a Natural Product (NP) pathway probability score > 0.8 and 309 of them (41%) were specific to each group as represented here.

S5. Annotated Base Peak Chromatograms of sponge crude extracts

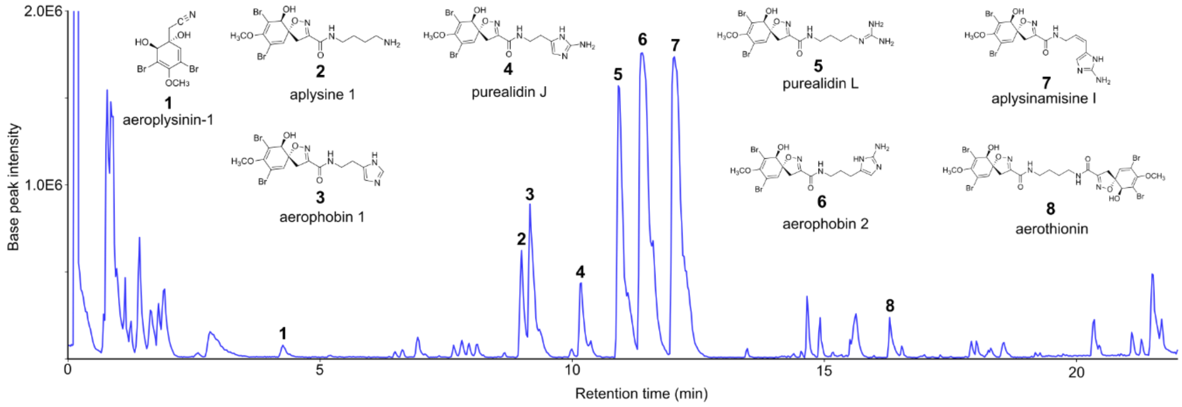

Figure S5.1. ***Aplysina cavernicola*** Annotated Base Peak Chromatogram (positive electrospray ionization) of a representative crude extract with identified brominated alkaloids (confidence level 1).

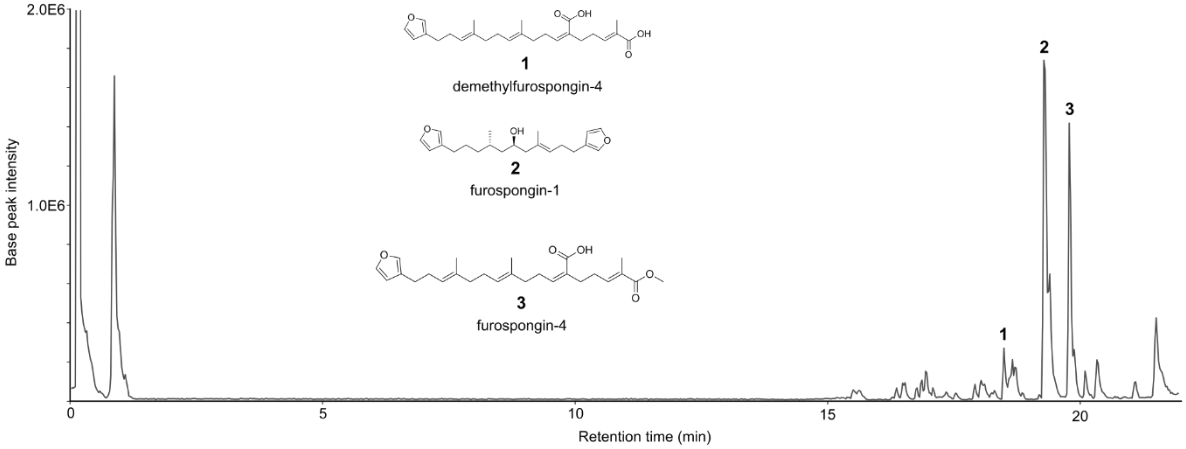

Figure S5.2. ***Spongia officinalis*** Annotated Base Peak Chromatogram (positive electrospray ionization) of a representative crude extract with its most abundant furanoterpenoids (confidence level 2b).

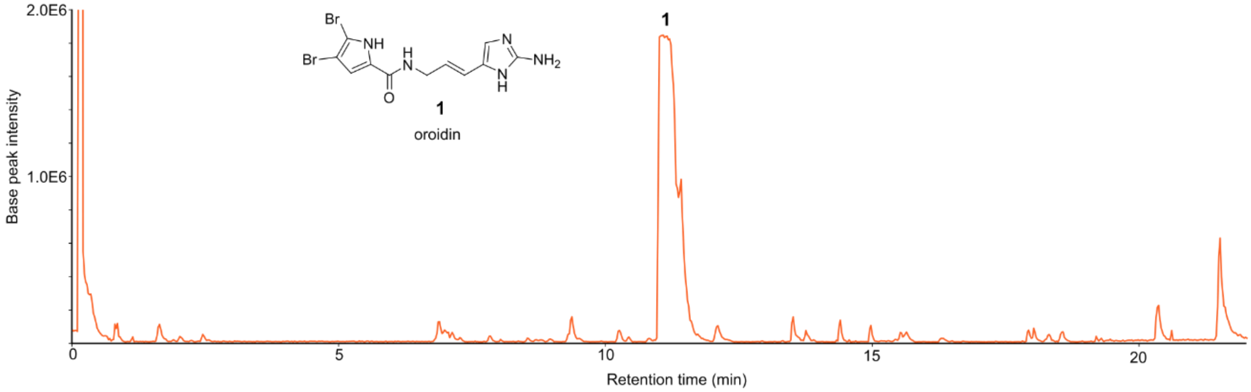

Figure S5.3. ***Agelas oroides*** Annotated Base Peak Chromatogram (positive electrospray ionization) BPC of a representative crude extract with oroidin its major brominated alkaloid (confidence level 1).

S6. MS data processing and annotated MS^2 spectra of reproducibly detected EMs

All MS^2 data were acquired with a Bruker Impact II (ESI-Q-ToF) using positive electrospray ionization (ESI+) as follows: nebulizer gas N_2 at 3.5 bar; dry gas at 12 $L.min^{-1}$, capillary temperature at 200°C and voltage at 4500 V. MS/MS acquisition mode was set with a scan rate of 8 Hz (full scan from 50-1200 *m/z*), and a mixed collision energy (CE) at 20-40 eV (50% time at each collision energy, stepping mode).

UHPLC-MS data processing Following their calibration, the acquired MS data were converted to the open format *.mzXML using MSConvert (Proteowizard)[7] and further processed on MZmine 3.2.8[8] for feature detection, as follows: (1) mass detection (centroid, MS1, noise level 1E4, and MS2 noise level 0), (2) ADAP chromatogram builder52 (2 scans, group intensity threshold 3E2, minimum highest intensity 3E2, m/z tolerance 10 ppm), (3) chromatogram resolving (baseline resolver: minimum peak height 1E4, peak duration range 0.0-2.0 min, baseline level 1E3, RT range for MS2 scan pairing 0.03 min, number of data point 2), (4) isotopic grouper (13C isotope filter: m/z tolerance 10 ppm, RT tolerance 0.1 min, representative isotope most intense), (5) join aligner (m/z tolerance 10 ppm, weight for m/z 75%, weight for RT 25%, RT tolerance 0.1 min), (6) feature list rows filter (RT 0.5-22.0 min, and keep only peaks with MS2 scans). MetaCorrelate (RT tolerance 0.03 min, minimum height 0, intensity correlation threshold 1E4, correlation grouping: measure PEARSON, minimum feature shape correlation 85%). Features detected only in MeOH were deleted. The generated MS2 feature list contained 2248 signals.

S6.1. *Aplysina cavernicola*

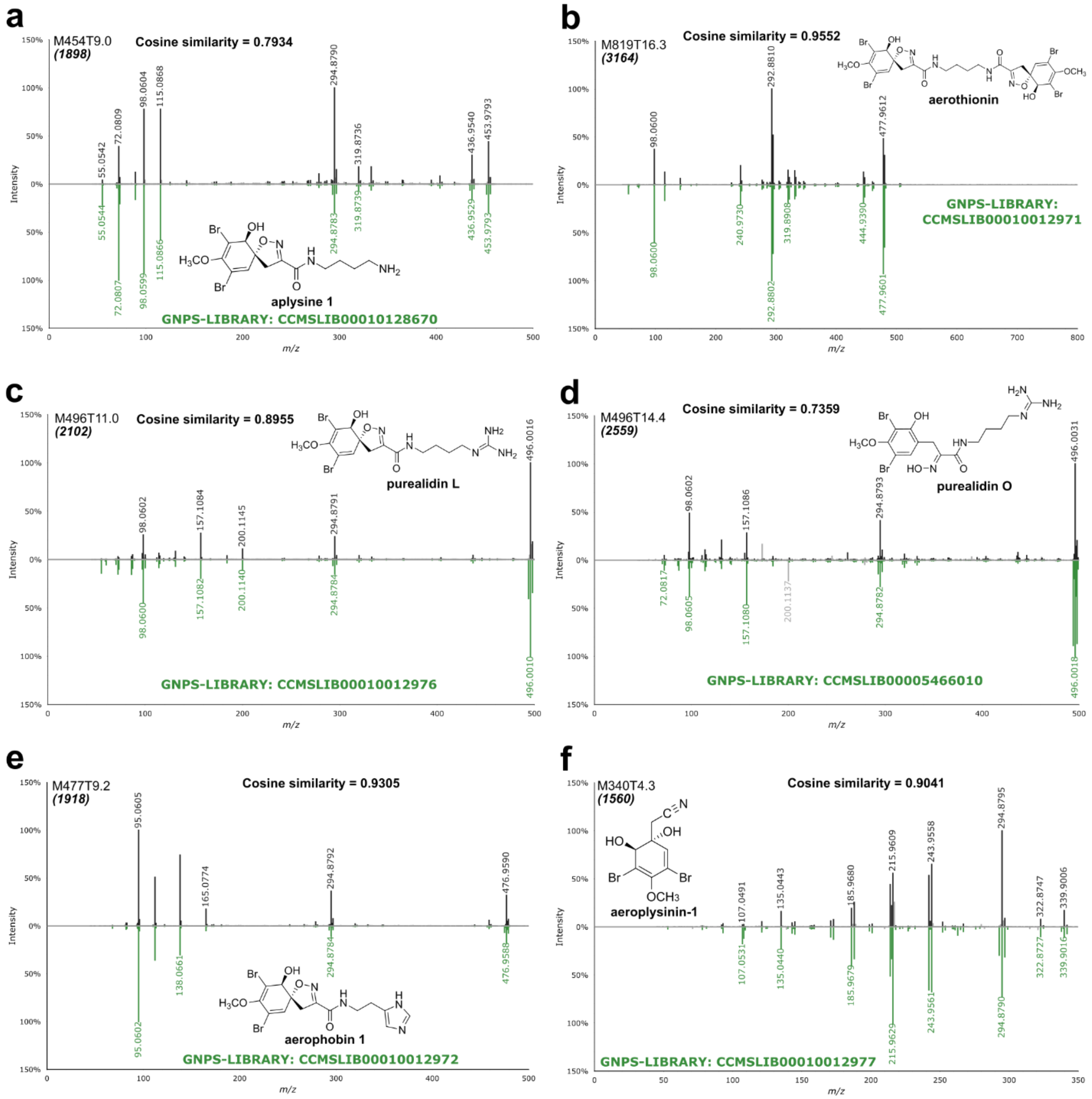

Figure S6.1.1. GNPS-library mirror image of MS^2 spectra of identified brominated alkaloids.

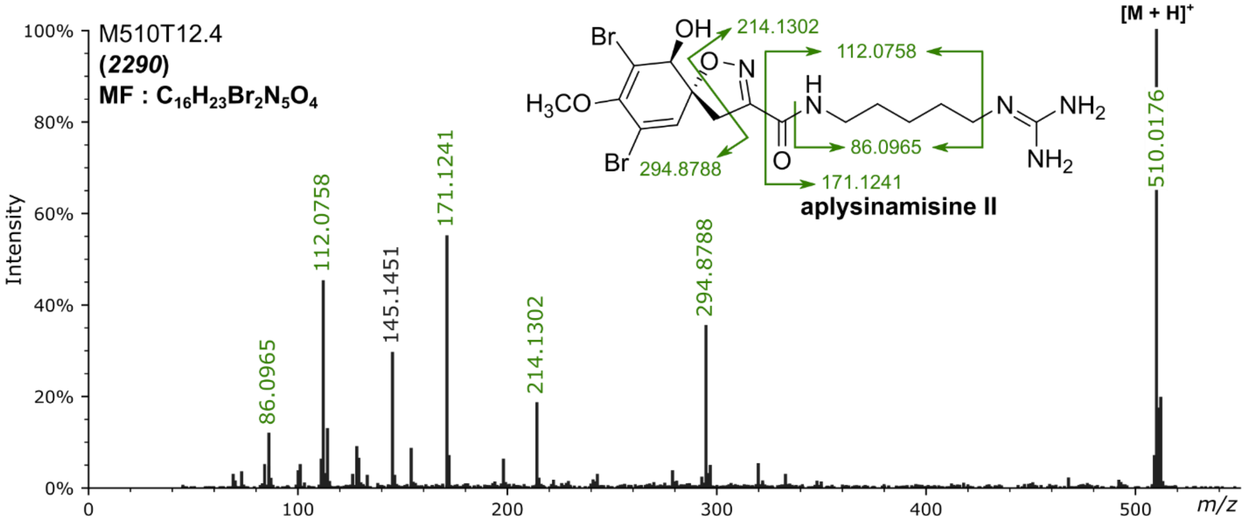

Figure S6.1.2. MS^2 spectrum of putative aplysinamisine II.

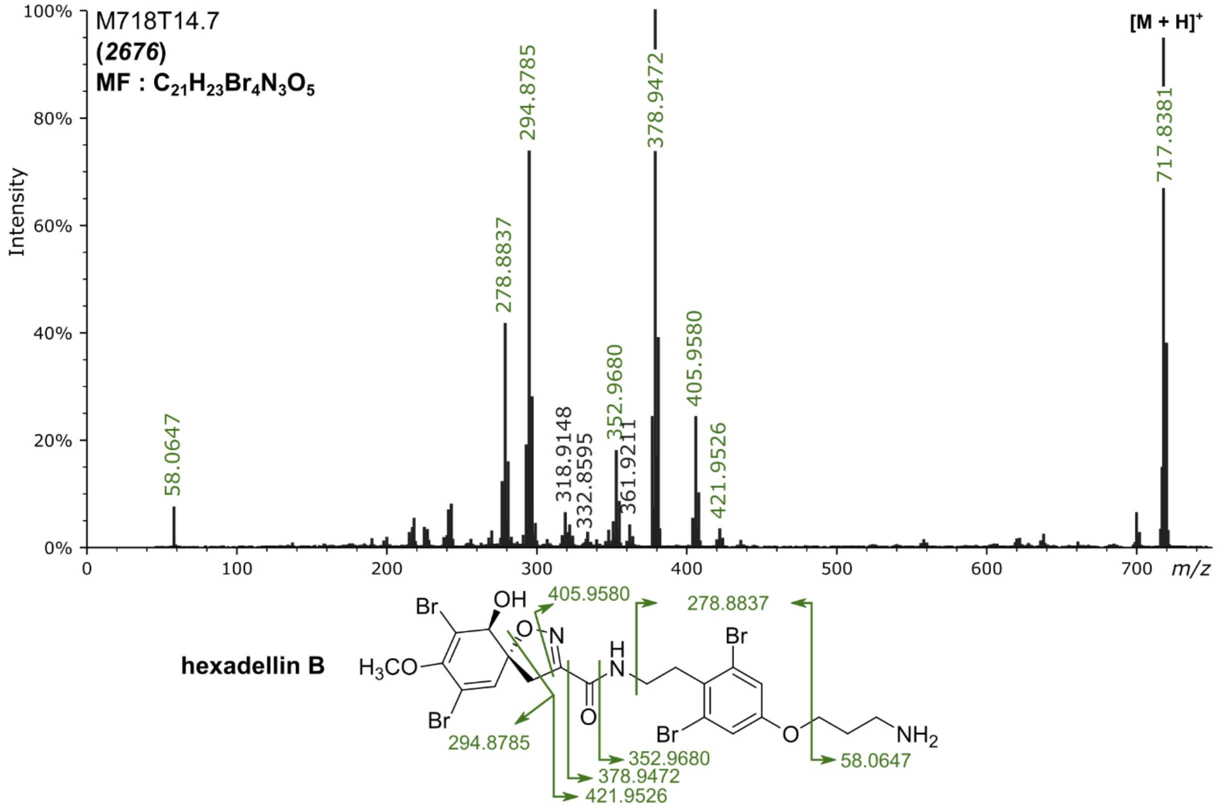

Figure S6.1.3. MS^2 spectrum of putative hexadellin B**.**

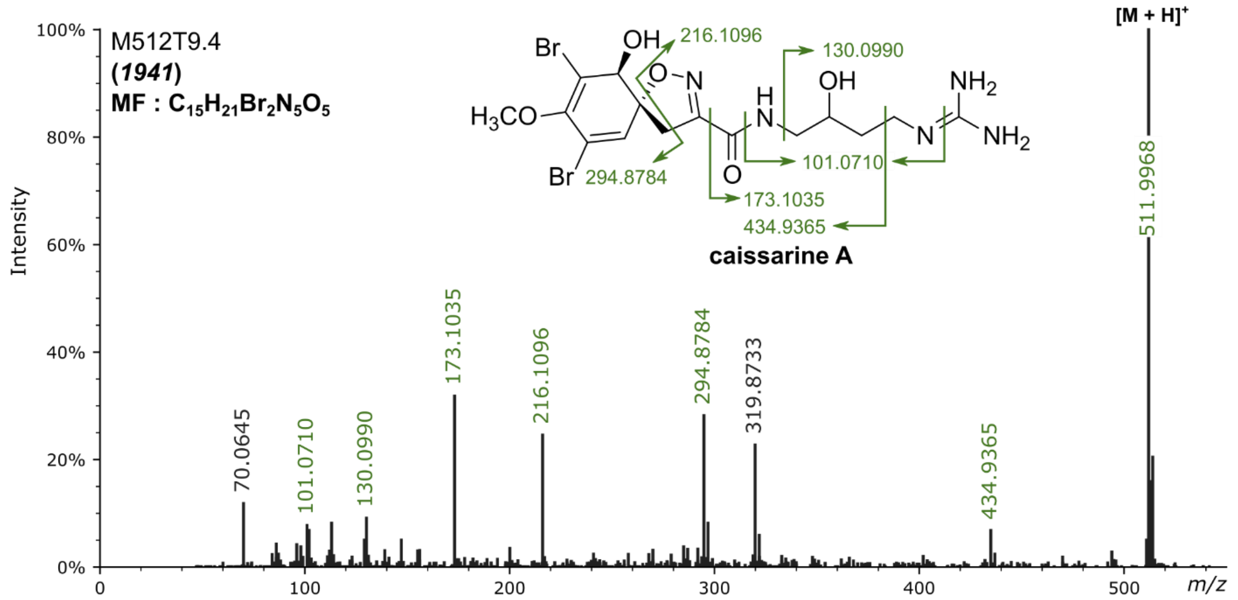

Figure S6.1.4. MS^2 spectrum of putative caissarine A.

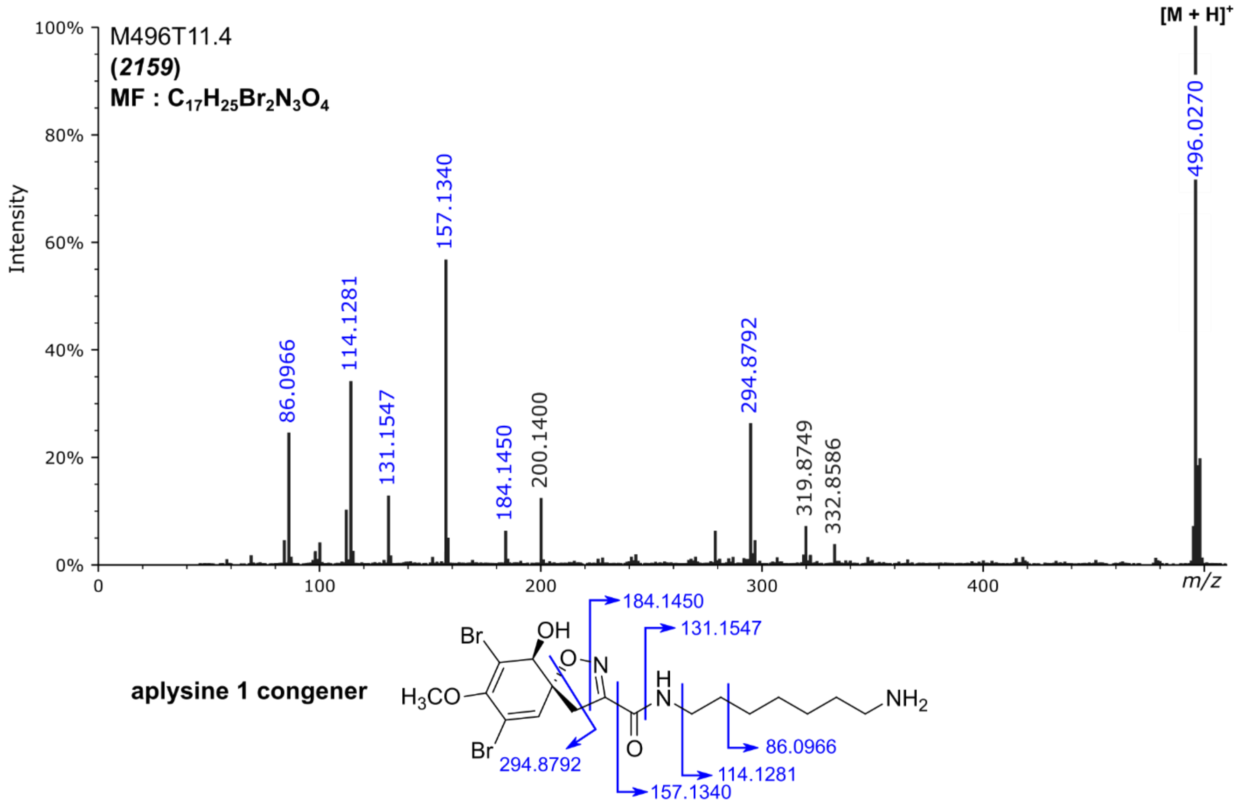

Figure S6.1.5. MS^2 spectrum of putative aplysine 1 congener M496T11.4.

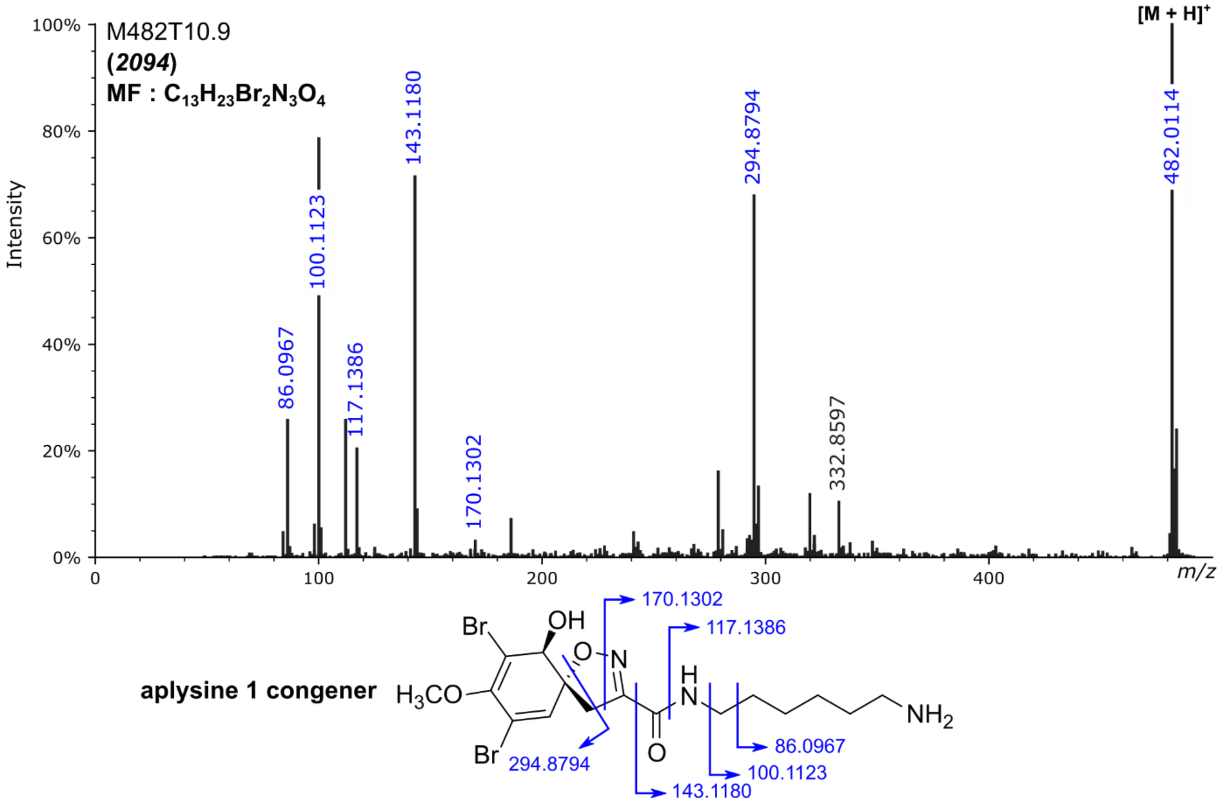

Figure S6.1.6. MS^2 spectrum of putative aplysine 1 congener M482T10.9.

Table S6.1. MS data of identified or annotated exometabolites found in *Aplysina cavernicola* crude extracts and reproducibly detected as exometabolites

Common Name	Code (Cluster index)[a]	RT (min)	Molecular formula	Ion status	Measured[b]	Calculated	MS² fragments & intensities[c]		ID[d]
					m/z				
Aeroplysinin-1	M340T4.3 (1560)	4.28	$C_9H_9Br_2NO_3$	$[M + H]^+$	337.9014	337.9022	121.0282<br>135.0443<br>187.9658<br>213.9631<br>241.9578<br>294.8795	5.3<br>16.0<br>25.6<br>44.2<br>53.5<br>100.0	1
Methyl acetamide of aeroplysinin-1	M394T4.3 (1563)	4.3	$C_{10}H_{13}Br_2NO_4$	$[M + Na]^+$	391.9105	391.9104	282.9627<br>334.8713<br>392.9110	16.6<br>7.8<br>5.8	3
-	M269T7.7 (1763)	7.65	$C_{18}H_{28}Br_2N_6O_3$	$[M + 2H]^{2+}$	268.0376	268.0368	72.0818<br>98.0604<br>131.1291<br>157.1090<br>184.1205<br>264.8652<br>362.9536<br>406.9417	58.1<br>90.3<br>58.1<br>100.0<br>45.2<br>37.1<br>48.4<br>21.0	3
Aplysine 1	M454T9.0 (1898)	9.0	$C_{14}H_{19}Br_2N_3O_4$	$[M + H]^+$	451.9813	451.9815	55.0542<br>72.0809<br>98.0604<br>115.0868<br>294.8790<br>319.8736<br>436.9540	4.0<br>38.9<br>77.8<br>77.8<br>100.0<br>17.8<br>30.0	1
Aerophobin 1	M477T9.2 (1918)	9.2	$C_{15}H_{16}Br_2N_4O_4$	$[M + H]^+$	474.9611	476.9611	95.0605<br>112.0872<br>138.0663<br>165.0774<br>294.8792	100.0<br>50.7<br>73.9<br>17.4<br>36.2	1
Caissarine A	M512T9.4 (1941)	9.4	$C_{15}H_{21}Br_2N_5O_5$	$[M + H]^+$	509.9977	509.9982	70.0645<br>101.0710<br>130.0990<br>173.1035<br>216.1096<br>294.8784<br>319.8733<br>434.9365	11.8<br>7.7<br>9.1<br>31.8<br>24.5<br>28.2<br>22.7<br>6.8	2b
Caissarine A analogue	M526T10.0 (1996)	10.0	$C_{16}H_{23}Br_2N_5O_5$	$[M + H]^+$	524.0122	524.0139	84.0805<br>130.0985<br>161.1400<br>169.1082<br>187.1191<br>214.1303<br>230.1264<br>294.8790	8.7<br>10.0<br>12.6<br>13.9<br>33.5<br>8.7<br>28.3<br>24.8	3
Aplysine 1 congener 1	M482T10.9 (2094)	10.9	$C_{16}H_{23}Br_2N_3O_4$	$[M + H]^+$	480.0124	480.0128	86.0967<br>100.1123<br>117.1386<br>143.1180<br>170.1302<br>294.8794<br>332.8597	25.7<br>78.6<br>20.4<br>71.4<br>3.1<br>67.9<br>10.4	3
Purealidin L	M496T11.0 (2102)	10.93	$C_{15}H_{21}Br_2N_5O_4$	$[M + H]^+$	494.0037	494.0033	98.0602<br>114.1029<br>131.1293<br>157.1084<br>200.1145<br>294.8791	25.5<br>6.4<br>8.5<br>27.3<br>10.9<br>23.6	1
Aplysine 1 congener 2	M496T11.4 (2159)	11.4	$C_{17}H_{25}Br_2N_3O_4$	$[M + H]^+$	494.0269	494.0285	86.0966<br>114.1281<br>131.1547<br>157.1340<br>184.1450<br>200.1400<br>294.8792	24.3<br>33.9<br>12.6<br>56.5<br>6.1<br>12.2<br>26.1	3

Common Name	Code (Cluster index)[a]	RT (min)	Molecular formula	Ion status	Measured[b]	Calculated	MS² fragments & intensities[c]		ID[d]
					m/z				
-	M552T11.8 (2214)	11.8	$C_{17}H_{21}Br_2N_5O_6$	$[M + H]^+$	549.9930	549.9931	96.0446<br>138.0664<br>155.0926<br>181.0723<br>208.0836<br>294.8795<br>519.9669	5.8<br>100.0<br>9.2<br>53.1<br>16.2<br>6.5<br>12.3	3
Aplysinamisine II	M510T12.4 (2290)	12.4	$C_{16}H_{23}Br_2N_5O_4$	$[M + H]^+$	508.0198	508.0190	86.0965<br>112.0758<br>171.1241<br>214.1302<br>294.8788	11.8<br>45.1<br>54.9<br>18.4<br>35.3	2b
Purealidin O	M496T14.4 (2559)	14.36	$C_{15}H_{21}Br_2N_5O_4$	$[M + H]^+$	494.0035	494.0033	98.0602<br>114.1028<br>131.1294<br>157.1086<br>294.8793	48.7<br>10.5<br>20.8<br>28.2<br>41.0	2b
Hexadellin B	M718T14.7 (2676)	14.7	$C_{21}H_{23}Br_4N_3O_5$	$[M + H]^+$	713.8431	713.8444	278.8837<br>294.8785<br>352.9580<br>361.9211<br>378.9472<br>405.9580	41.6<br>73.7<br>17.9<br>4.1<br>100.0<br>24.2	2b
-	M483T15.0 (2827)	15.0	$C_{15}H_{18}Br_2N_2O_6$	$[M + H]^+$	480.9590	480.9604	86.0598<br>101.0597<br>112.0391<br>130.0500<br>144.0659<br>294.8782<br>319.8717<br>332.8574<br>347.8775	45.7<br>23.9<br>18.5<br>78.3<br>26.1<br>100.0<br>17.6<br>23.9<br>80.4	3
Aerothionin	M819T16.3 (3164)	16.3	$C_{24}H_{26}Br_4N_4O_8$	$[M + H]^+$	814.8529	814.8557	98.0603<br>115.0870<br>294.8790<br>479.9596	33.8<br>12.8<br>100.0<br>50.7	1

[a]The Cluster index identifies nodes in the GNPS molecular network, the most intense peak of the isotopic pattern was used for feature codification. [b]The monoisotopic peak was selected for molecular formula determination. [c]Major MS² fragments and their intensities were selected using the GNPS Metabolomics USI tool. [d]Confidence level of metabolite identification according to Schymanski *et al.* 2014.[9]

S6.2. *Spongia officinalis*

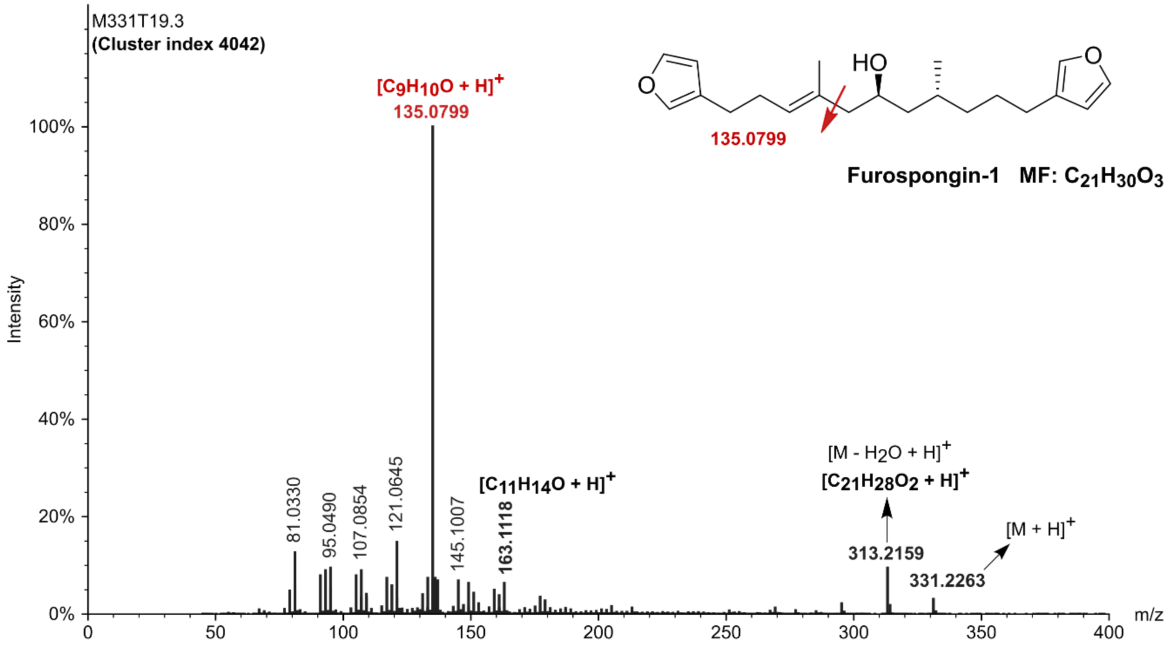

Figure S6.2.1. Annotated MS^2 spectrum of putative furospongin-1.

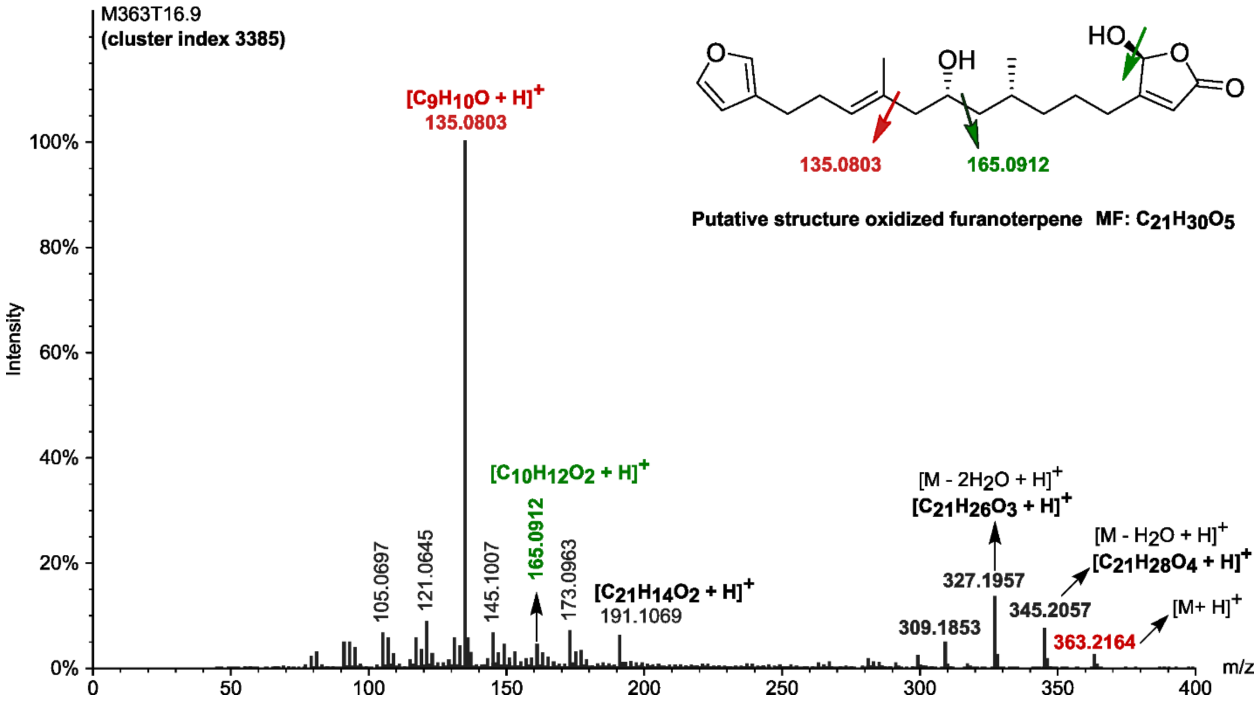

Figure S6.2.2. Annotated MS^2 spectrum of putative oxidized furospongin derivative.

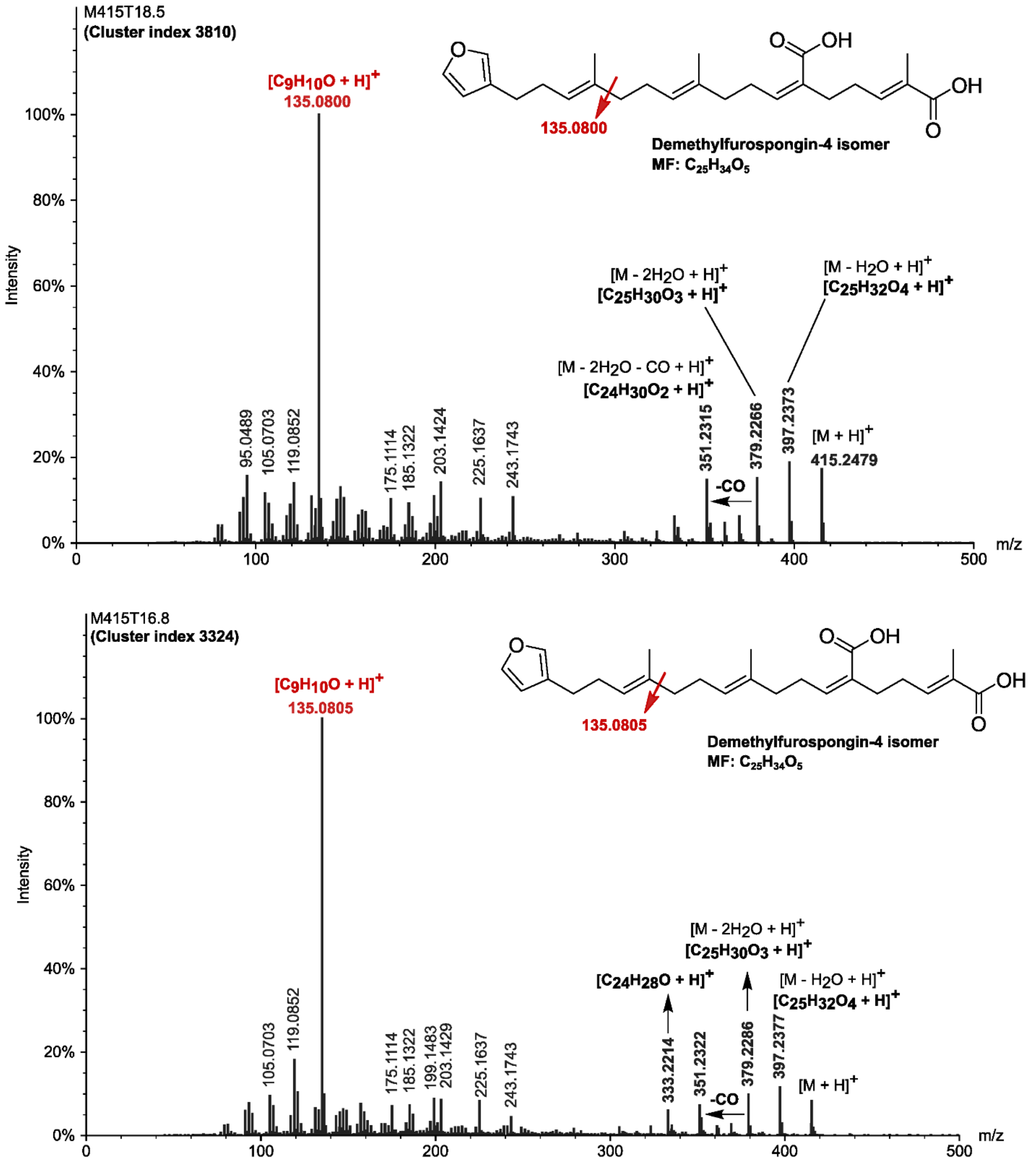

Figure S6.2.3. Annotated MS^2 spectra of putative demethylfurospongin-4 isomers.

The MS^2 spectra for the proposed demethylfurospongin-4 isomers, agree with data previously published by Bauvais *et al*. 2017.[10] Demethylfurospongin-4 is one of the most abundant furanoterpene in *Spongia officinalis* extract.[10] Double bond isomerism could explain the difference in retention time.

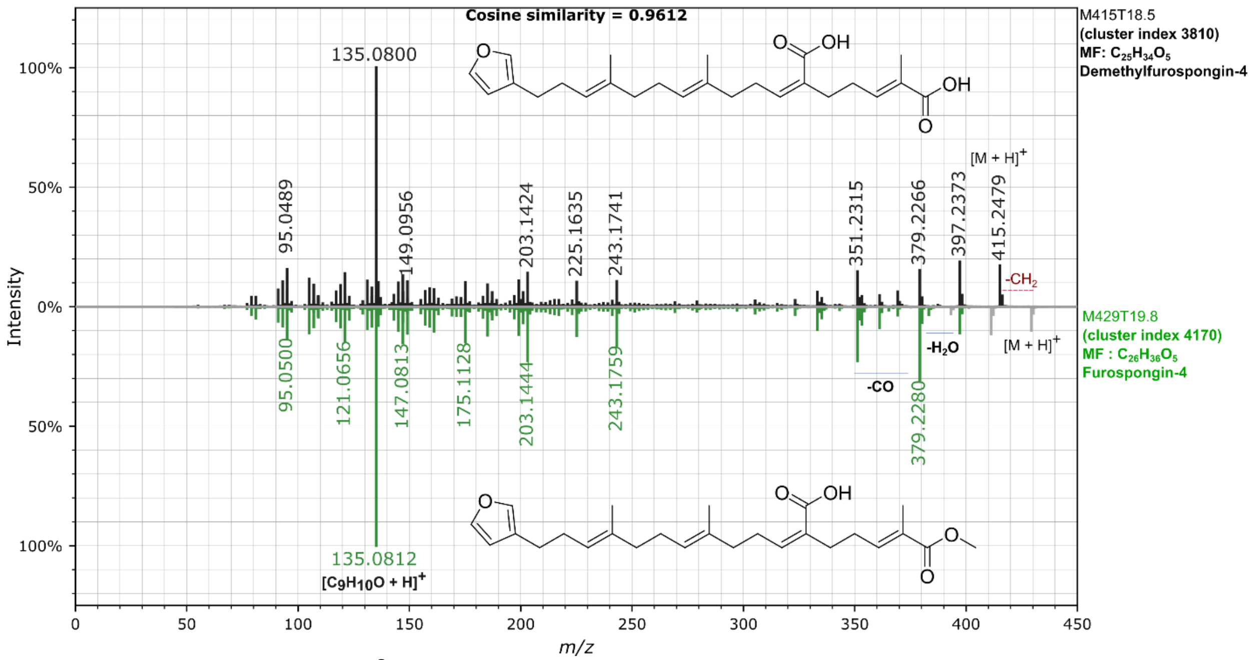

Figure S6.2.4. Comparative MS[2] spectra of furospongin-4 and demethylfurospongin-4 isomer.

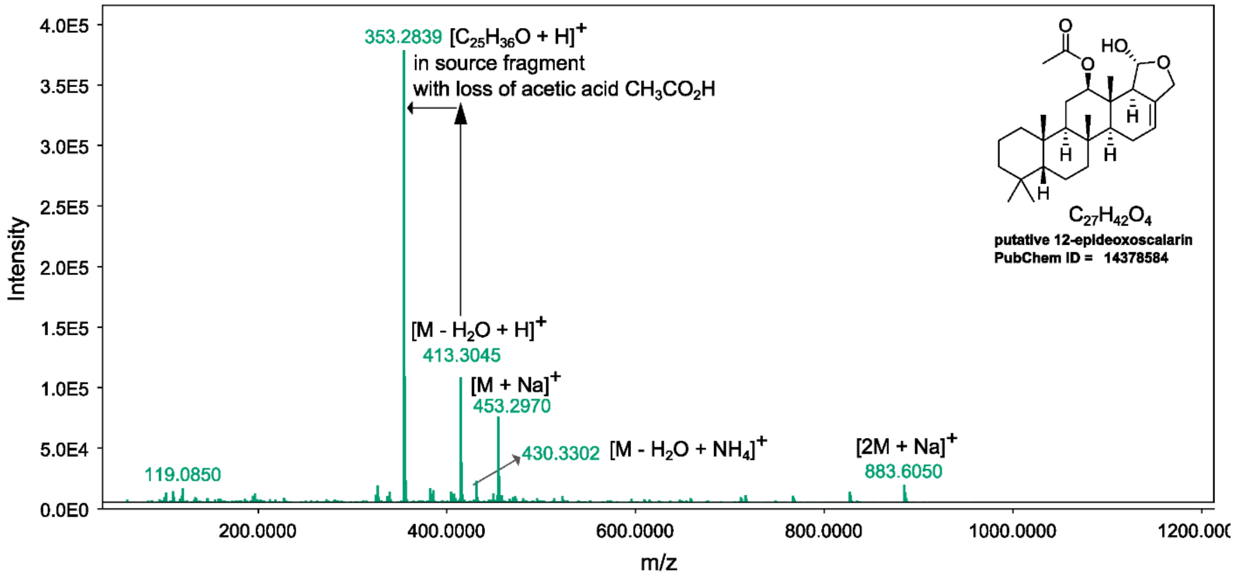

Figure S6.2.5. Annotated MS[1] spectra of M353T21.1 as putative 12-epideoxoscalarin.

The described MS[1] spectrum for the proposed scalarane sesterterpene, 12-epideoxoscalarin, agrees with data previously published (Noyer *et al.* 2011).[11]

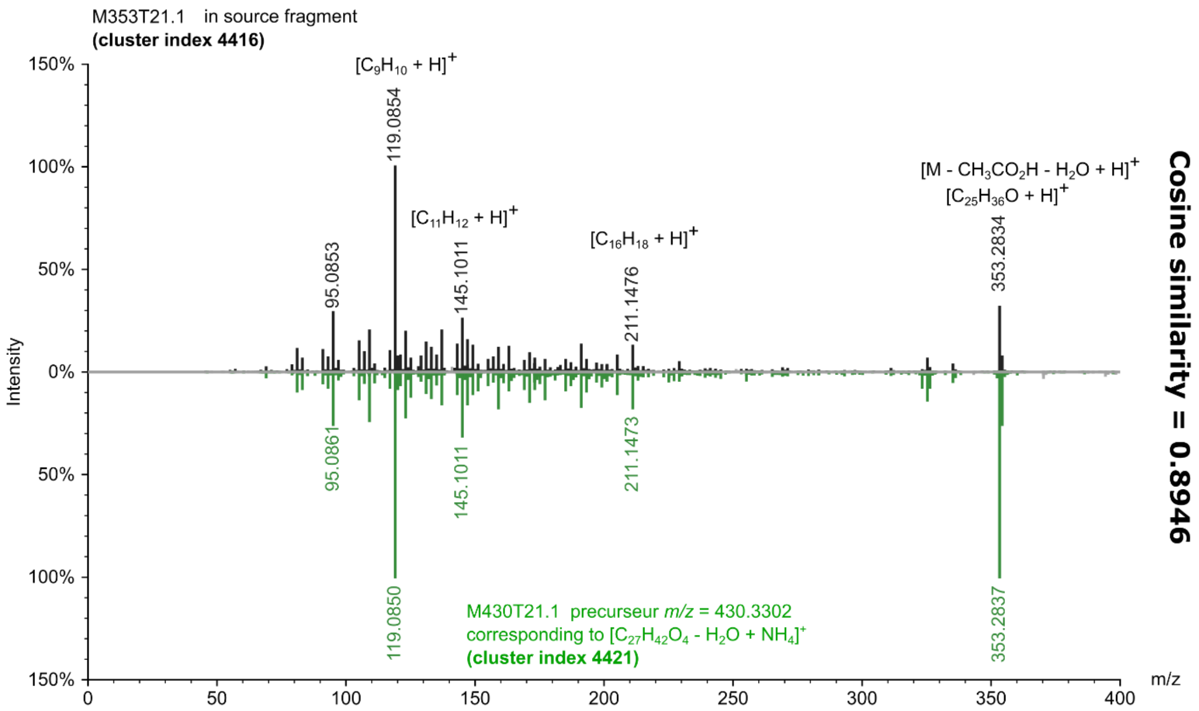

Figure S6.2.6. Comparative MS^2 spectra of M353T21.1 and M430T21.1.

Annotated MS^2 spectrum for the proposed scalarane sesterterpene, 12-epideoxoscalarin detected as *Spongia officinalis* exometabolite.

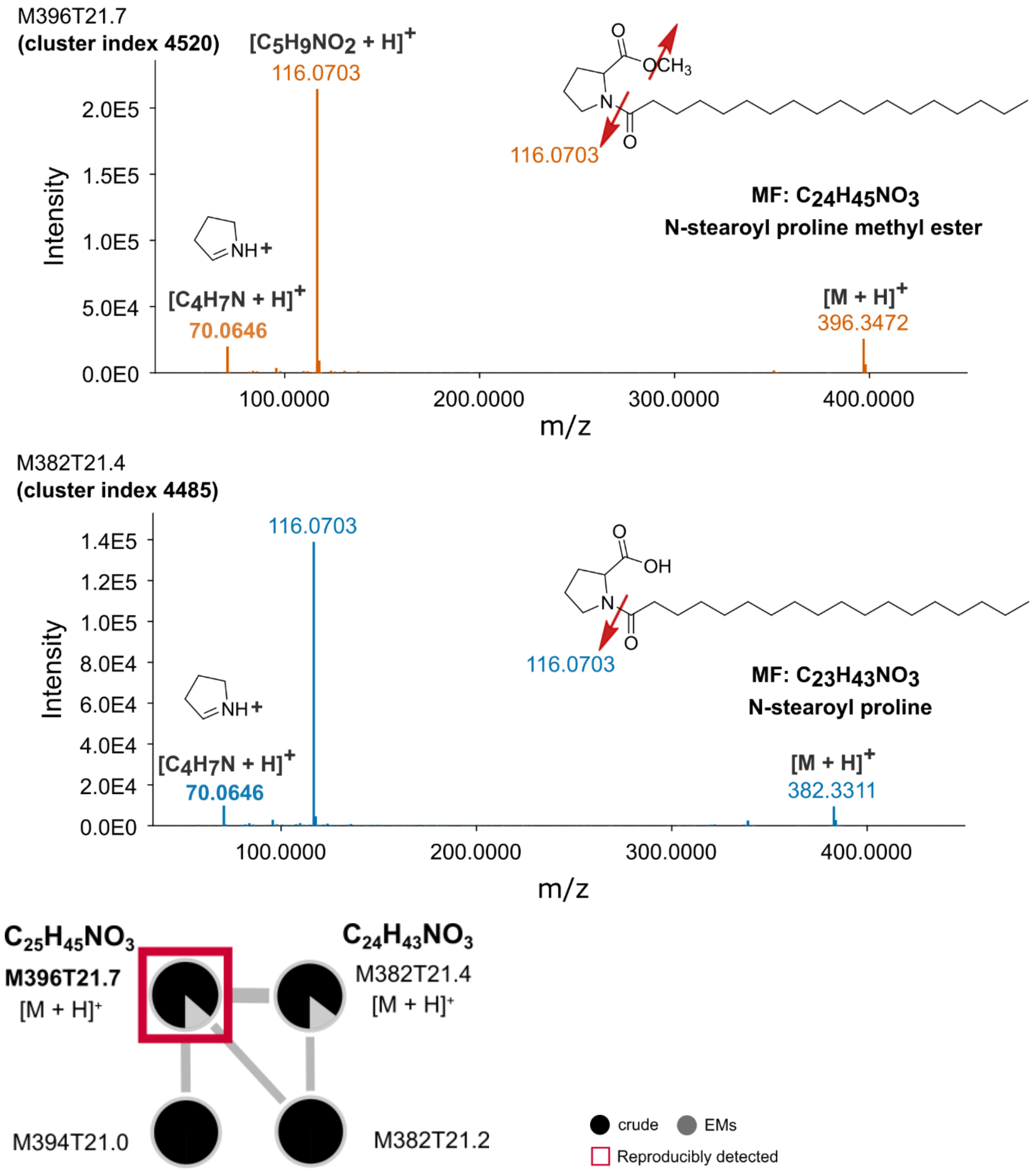

Figure S6.2.7. Annotated MS^2 spectrum of M396T21.7 (putative N-stearoyl proline methyl ester).

Table S6.2. MS data of putatively identified exometabolites found in *Spongia officinalis* crude extract and reproducibly detected as exometabolite

**Common Name**	**Code** **(Cluster index)[a]**	**RT (min)**	**Molecular formula**	**Ion status**	**Measured**	**Calculated**	**MS^2 fragments & intensities[b]**		**ID[c]**
					m/z				
Demethyl furospongin-4 isomer	M415T16.8 (3324)	16.8	$C_{25}H_{34}O_5$	$[M + H]^+$	415.2483	415.2479	105.0703<br>119.0852<br>**135.0805**<br>199.1483<br>379.2286<br>397.2377	9.4<br>18.1<br>100.0<br>8.7<br>9.7<br>11.5	2b
Oxidized furanoterpene	M363T16.9 (3385)	16.9	$C_{21}H_{30}O_5$	$[M + H]^+$	363.2168	363.2166	105.0697<br>121.0645<br>**135.0803**<br>145.1007165.0912<br>173.0963<br>327.1957<br>345.2057	6.5<br>8.7<br>100.0<br>6.5<br>6.5<br>7.0<br>13.5<br>7.4	3
Demethyl furospongin-4 isomer	M415T18.5 (3810)	18.5	$C_{25}H_{34}O_5$	$[M + H]^+$	415.2484	415.2479	95.0489<br>**135.0800**<br>203.1424<br>351.2315<br>379.2266<br>397.2373<br>415.2479	15.6<br>100.0<br>14.1<br>14.7<br>15.2<br>18.8<br>17.2	2b
Furospongin-1	M331T19.3 (4042)	19.3	$C_{21}H_{30}O_3$	$[M + H]^+$	331.2271	331.2268	81.0330<br>95.0490<br>107.0854121.0645<br>**135.0799**<br>313.2159	12.6<br>9.5<br>8.9<br>14.7<br>100.0<br>9.5	2b
-	M353T20.1 (4229)	20.1	$C_{21}H_{36}O_4$	$[M + H]^+$	353.2676	353.2686	167.1432<br>168.1463	100.0<br>10.9	3
12-epideoxoscalarin	M353T21.1 (4416)	21.1	$C_{27}H_{42}O_4$	$[M - CH_3CO_2H - H_2O + H]^+$<br>$[M - H_2O + H]^+$<br>$[M + Na]^+$	353.2834<br>413.3045<br>453.2970	353.2844<br>413.3055<br>453.2975	95.0853<br>109.1010<br>119.0854<br>137.1321<br>145.1011<br>353.2834	28.9<br>20.0<br>100.0<br>20.0<br>25.8<br>31.6	2b
N-stearoyl proline methyl ester	M396T21.7 (4520)	21.7	$C_{24}H_{45}NO_3$	$[M + H]^+$	396.3472	396.3472	70.06461116.0703<br>396.3472	9.5<br>100.0<br>12.4	3

[a]The Cluster index identifies nodes in the GNPS molecular network, the most intense peak of the isotopic pattern was used for feature codification. [b]Major MS^2 fragments and their intensities were selected using the GNPS Metabolomics USI tool. [c]Confidence level of metabolite identification according to Schymanski *et al.* 2014.[9]

S6.3. ***Agelas oroides***

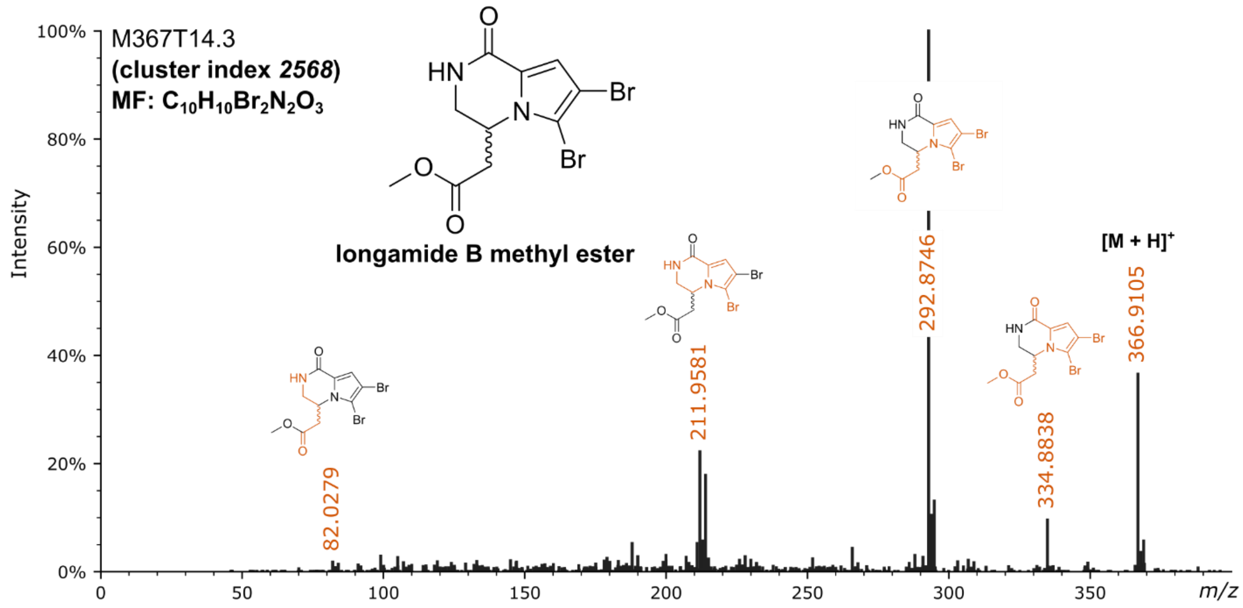

Figure S6.3.1. MS^2 spectrum of putative Longamide B methyl ester.

The proposed structure for M367T14.3 is in agreement with previously reported data.[12] The corresponding cluster index is between brackets (see also table below).

Table S6.3. MS data of putatively identified exometabolites found in *Agelas oroides* crude extract and reproducibly detected as exometabolite

Common Name	Code (Cluster index)[a]	RT (min)	Molecular formula	Ion status	Measured[b] (m/z)	Calculated (m/z)	MS^2 fragments & intensities[c]		ID[d]
Longamide B	M353T12.3 (2284)	12.3	$C_9H_8Br_2N_2O_3$	$[M + H]^+$	352.8955	352.8979	82.0283<br>211.9583<br>292.8744<br>334.8838	3.0<br>23.3<br>91.7<br>5.4	2b
Longamide B methyl ester	M367T14.3 (2568)	14.3	$C_{10}H_{10}Br_2N_2O_3$	$[M + H]^+$	364.9120	364.9131	82.0279<br>211.9581<br>292.8746<br>334.8838	1.8<br>22.2<br>100.0<br>9.6	2b

[a]The Cluster index identifies nodes in the GNPS molecular network, the most intense peak of the isotopic pattern was used for feature codification. [b]The monoisotopic peak was selected for molecular formula determination. [c]Major MS^2 fragments and their intensities were selected using the GNPS Metabolomics USI tool. [d] Confidence level of metabolite identification according to Schymanski *et al.* 2014.[9]

S7. ^{1}H NMR spectra of sponge EM extracts (EXP2)

Acquisitions parameters:

NMR analyses were performed on a Bruker Avance II+ spectrometer at 600 MHz equipped with a TCI Cryoprobe at 300 K. The probe frequency was tuned and impedance matched before each acquisition. The acquired spectra were processed using Topspin 4.3 NMR software package (Bruker BioSpin). Samples were exactly weighed and diluted in 100 µL of deuterated MeOH (CD_3OD 99.95%-d, MagniSolv, Merck) then transferred in 2 mm o.d. Match NMR tubes (Bruker # 67539).

The ^{1}H NMR acquisition parameters were set up as follows for all EM extracts:

Total duration of acquisition: 27 min

**Pulse program**	**zgcppr (1D sequence with presaturation of water signal)**
P1 (90 pulse)	6.59 µsec for AC_EM, 6.61 µsec for SO_EM, 6.90 µsec for AO_EM
O1P-SW	4.969 ppm (2982.09 Hz), SW:22 ppm
D1	3 sec
NS	384
RG	32
AQ	1.27 sec

P1: 90-degree transmitter high power pulse, SW: Spectral Width, O1P: Transmitter frequency offset (ppm), D1: relaxation delay (sec), NS: number of scans, RG: receiver gain, AQ: Acquisition time (sec).

S7.1. *Aplysina cavernicola*

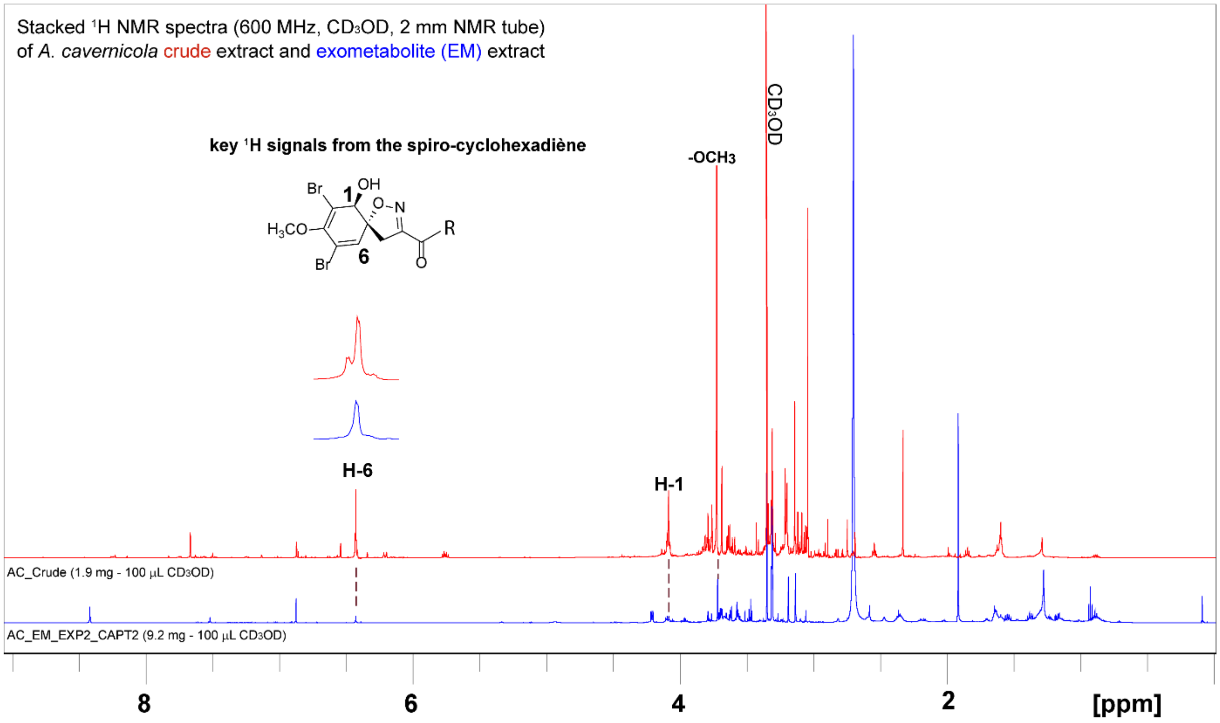

Figure S7.1.1. Stacked ^{1}H NMR spectra of *Aplysina cavernicola* crude extract with one EM extract.

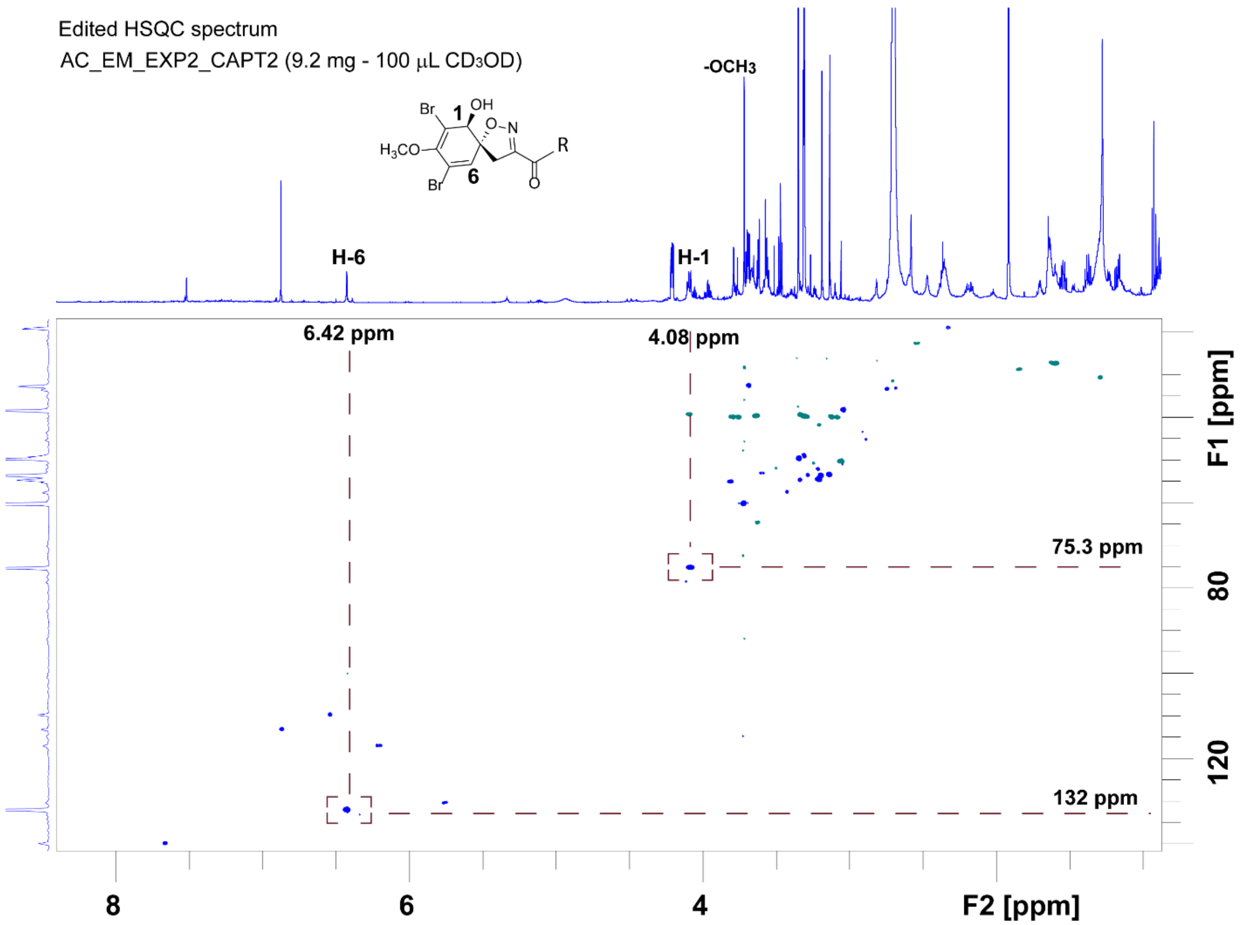

Figure S7.1.2. Key HSQC ^{1}H-^{13}C correlations, confirming the identity of H-6 and H-1, in agreement with previously published information (Mauduit *et al*. 2022, raw data accessible at https://zenodo.org/record/6375672)[135]

Table S7.1. 2D gHSQC NMR acquisition parameters (duration 2h56 min)

Pulse program	Hsqcedetgpsisp2.4 (NUSAmount = 25%) Non-uniform sampling
P1 (90 pulse)	6.59 usec
SW (O1P)	F2: 14 (5) ppm, F1: 165 (75) ppm
D1	1.5 sec
NS	100
DS	16
RG	2050
AQ	0.121 sec (F2), 0.005 sec (F1)

NUSAmount : Amount of sparse sampling set at 25%, P1: 90-degree transmitter high power pulse, SW: Spectral Width, O1P: Transmitter frequency offset (ppm), D1: relaxation delay (sec), NS: number of scans, DS: Dummy scans, RG: receiver gain, AQ: Acquisition time (sec), S/N: signal to noise ratio.

S7.2. *Spongia officinalis*

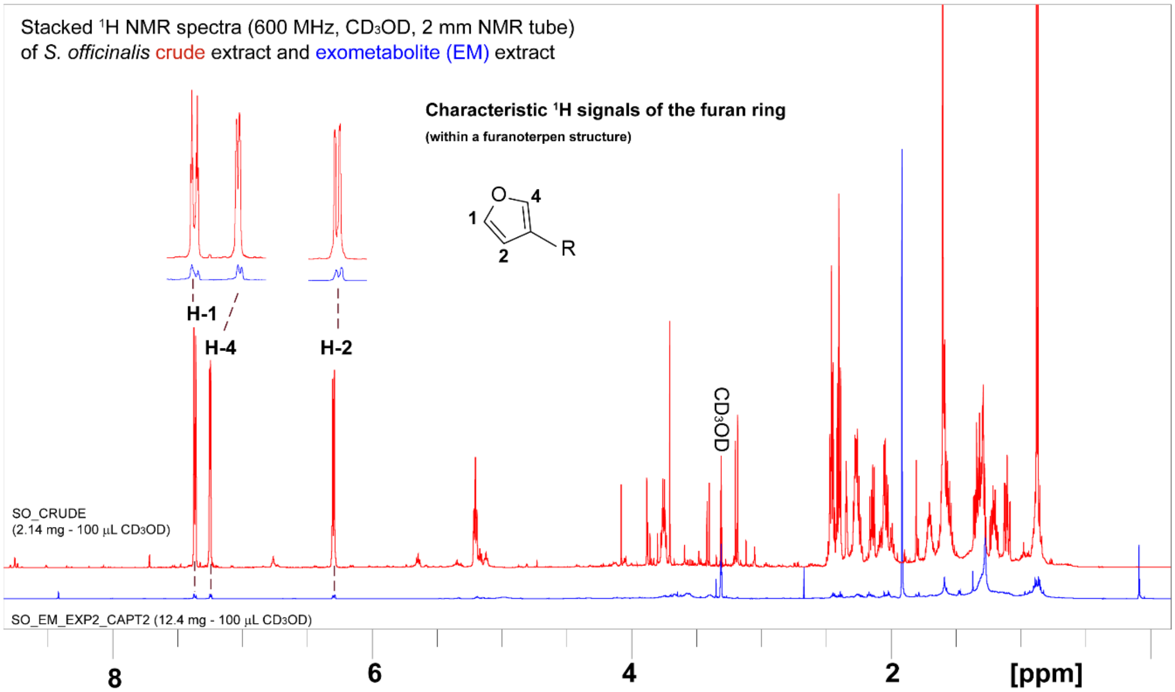

Figure S7.2.1 Stacked ^{1}H NMR spectra of *Spongia officinalis* crude extract with one EM extract. The ^{1}H chemical shifts from the furan ring are in agreement with previously published data in Bauvais *et al.* 2017.[10] See also information at Natural Product NMR-DB "CH-NMR-NP" (https://www.j-resonance.com/en/nmrdb/).

S7.3. *Agelas oroides*

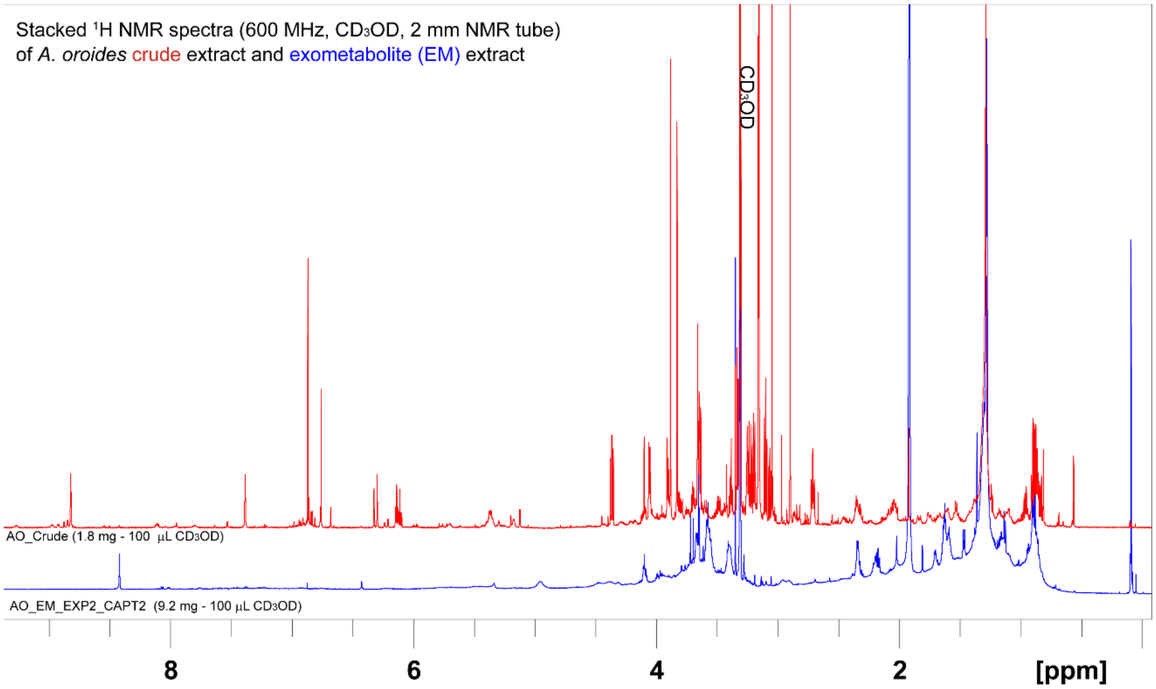

Figure S7.3.1 Stacked ^{1}H NMR spectra of *Agelas oroides* crude extract with one EM extract. Using the ^{1}H NMR set-up acquisition parameters, we could not observe any significant ^{1}H signal corresponding to the reproducibly released longamide B methyl ester in the NMR spectrum of *A. oroides* EM extracts.

S7.4. Stacked ^{1}H NMR spectra of all EM extracts (EXP2)

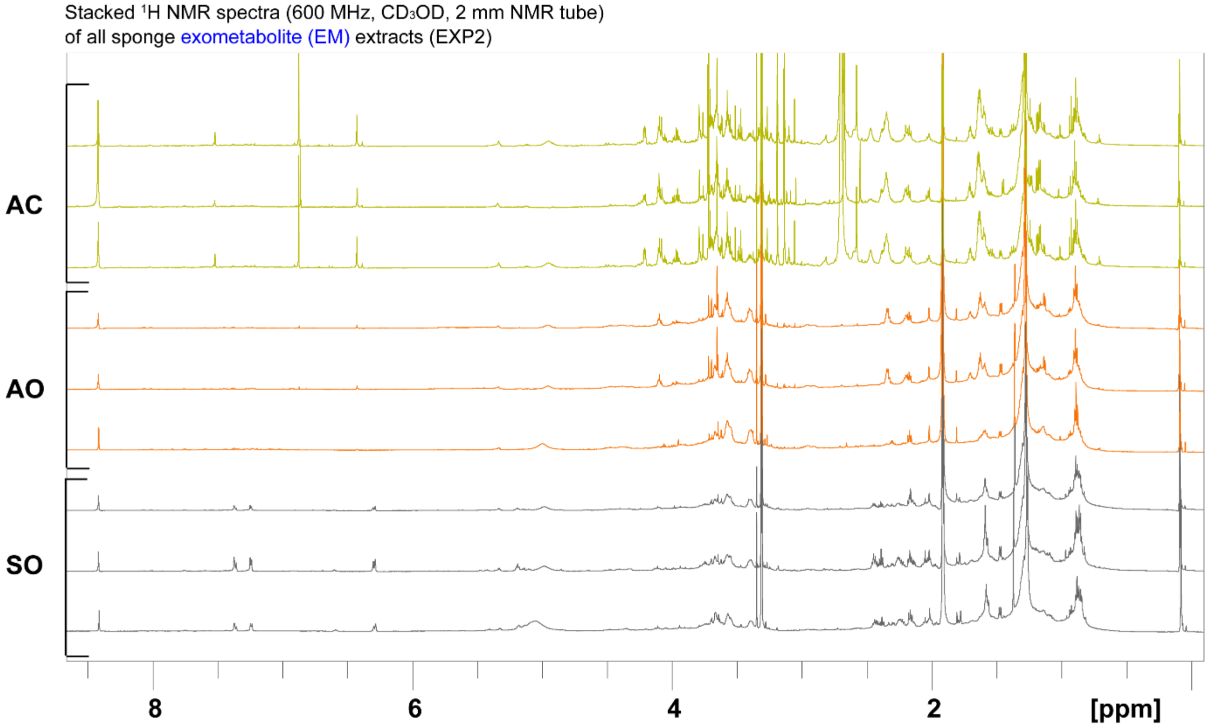

Figure S7.4. Stacked ^{1}H NMR spectra of all EM extracts from EXP2. The figure shows that each averaged sponge EM extract showed unique ^{1}H NMR fingerprints. Characteristic NMR signals corresponding to sponge specialized metabolites were observed for *A. cavernicola* and *S. officinalis*. For both sponges, several metabolites with a common core structure were identified in all EM extracts. Consequently, these metabolites collectively contributed to detectable ^{1}H signals in the NMR spectrum. For *A. oroides*, the only detection of longamide B methyl ester as reproducible EM poses a challenge for its detection by NMR.

REFERENCES:

1. Berlinck, R. G. S. *et al.* The isolation of water-soluble natural products – challenges, strategies and perspectives. *Nat. Prod. Rep.* 10.1039.D1NP00037C (2021)
2. Dittmar, T., Koch, B., Hertkorn, N. & Kattner, G. A simple and efficient method for the solid-phase extraction of dissolved organic matter (SPE-DOM) from seawater. *Limnol. Oceanogr. Methods* **6**, 230–235 (2008).
3. Petras, D. *et al.* High-Resolution Liquid Chromatography Tandem Mass Spectrometry Enables Large Scale Molecular Characterization of Dissolved Organic Matter. *Front. Mar. Sci.* **4**, (2017).
4. Roué, M., Darius, H. & Chinain, M. Solid Phase Adsorption Toxin Tracking (SPATT) Technology for the Monitoring of Aquatic Toxins: A Review. *Toxins* **10**, 167 (2018).
5. Bogdanov, A. *et al.* Small Molecule in situ Resin Capture – A Compound First Approach to Natural Product Discovery. 2023.03.02.530684 Preprint at https://doi.org/10.1101/2023.03.02.530684 (2023).
6. Vlachou, P. *et al.* Innovative Approach to Sustainable Marine Invertebrate Chemistry and a Scale-Up Technology for Open Marine Ecosystems. *Mar. Drugs* **16**, 0152–0152 (2018).
7. Chambers, M. C. *et al.* A cross-platform toolkit for mass spectrometry and proteomics. *Nat. Biotechnol.* **30**, 918–920 (2012).
8. Schmid, R. *et al.* Integrative analysis of multimodal mass spectrometry data in MZmine 3. *Nat. Biotechnol.* 1–3 (2023)
9. Schymanski, E. L. *et al.* Identifying Small Molecules via High Resolution Mass Spectrometry: Communicating Confidence. *Environ. Sci. Technol.* **48**, 2097–2098 (2014).
10. Bauvais, C. *et al.* Furanoterpene Diversity and Variability in the Marine Sponge *Spongia officinalis*, from Untargeted LC–MS/MS Metabolomic Profiling to Furanolactam Derivatives. *Metabolites* **7**, 27 (2017).
11. Noyer, C., Thomas, O. P. & Becerro, M. A. Patterns of Chemical Diversity in the Mediterranean Sponge S*pongia lamella*. *PLoS ONE* **6**, e20844 (2011).
12. Freire, V. F. *et al.* Feature-Based Molecular Networking Discovery of Bromopyrrole Alkaloids from the Marine Sponge *Agelas dispar*. *J. Nat. Prod.* **85**, 1340–1350 (2022).
13. Mauduit, M. *et al.* Diving into the Molecular Diversity of *Aplysina cavernicola*'s Exometabolites: Contribution of Bromo-Spiroisoxazoline Alkaloids. *ACS Omega* **7**, 43068–43083 (2022).
