# Supplementary material for: In Situ Capture and Real-Time Enrichment of Marine Chemical Diversity
Source: ACS Cent Sci. 2023 Nov 8;9(11):2084–95. doi: 10.1021/acscentsci.3c00661 (PMC10683479; doi:10.1021/acscentsci.3c00661)
Supplement: Supplementary file 2 — oc3c00661_si_002.pdf [file oc3c00661_si_002.pdf]

oc-2023-00661c.R1

Name: Peer Review Information for "In Situ Capture and Real Time Enrichment of Marine Chemical Diversity"

First Round of Reviewer Comments

Reviewer: 1

Comments to the Author

In this work, Mauduit et al have developed an instrument to concentrate metabolites excreted by three species of sponges, which are known producers of natural products and applied advanced dereplication strategies to describe the chemical diversity captured using untargeted metabolomics. With the advancement in mass spectrometry instruments and data analytics, development of such methods is significant as it will enable elucidation of chemical crosstalk between life forms in the ocean environments. The introduction is very well written and sets the stage for understanding why the development of this sampling instrument is significant. The study is well designed and executed with appropriate replicates and inclusion of crude extracts of sponge specimen for comparison. The confidence level for annotations is clearly stated and appropriate for this study. Few minor points are listed below which will improve the readability of the manuscript.

1. The authors should revise the first line of the abstract. The phrase: “its related ecosystem functions” appears to not fit in the sentence and can be elaborated upon.

2. Line 77 Page 4 (introduction section): “Therefore the idea of alternative SPE approaches.” The authors could consider citing “R. N. Tuttle et al., Applied and Environmental Microbiology; 2019: 85, e02830-02818 and the preprint titled “Small Molecule in situ Resin Capture – A Compound First Approach to Natural Product Discovery” by Paul Jensen lab here as well https://doi.org/10.1101/2023.03.02.530684

3. A small panel either in main text figure or as supporting figure to provide visual guide of replication and sample types collected would be useful (description in section titled in situ experiments under results). For example: For EXP-2, it appears that a sample was collected three times on each specimen, and a total of five specimens of each species were collected. Would this be total of 45 (3*5*3) samples for three species? This would then result in 45/5=9 extracts. Similarly, it appears a total of 15 crude extracts were generated, five for each species and they were pooled resulting in 15/5=3 crude extracts. A visual figure panel will assist in understanding of this scheme.

4. Methods section: “These captures, corresponding to EXP2, were done with all valves opened to obtain three separate DVB filters representing three technical replicates.” Was the data collected

separately on these technical replicates? I believe separately and that is why authors have 9 individual extracts (3 for each species). These details are hard to parse out as they are divided between methods and results.

5. Based on Fig. 3B: It appears that 16% (100- (36+20+10+8) of the features are displayed as a black box, which represents crude extracts. Authors can consider labeling 16% on black box to be consistent.

6. The brief explanation on the choice of divenylbenezene polymer as compared to other chemistries available to adsorb metabolites should be moved from methods to results section.

7. Line 192, Page 11: "The most polar EM aeroplysinin, was also found to be proportionally 25 times more abundant 193 in EM extract than in crude extracts." The crude extracts were generated using C18 SPE which is a different matrix than the DVB polymer used for enriching EMs from sea water. One is extracted from liquid material (10L) and other from solid material (1 g powder). While the presence and absence comparisons are straightforward, comparing abundances can be tricky due to differences in sample types and extraction procedures. These should be clarified as a discussion line while comparing the metabolite abundances between the two Or the authors can simply add a phrase "highlighting enrichment capacity of I-SMEL as they have done elsewhere in the manuscript.

8. Figure 4 is mistakenly labeled as Fig 41

9. Discussion: Page 16 line 258 "A possible explanation is that sponges do not…" As higher chemical diversity is detected in enriched samples via I-SMEL, some of the metabolites might not be detected in EMs (below detection limit or ionization repressed due to a co-luting metabolite not present in crude extract). This alternative explanation can also be added as the authors cannot rule out this possibility for a subset of detected features.

Reviewer: 2

Comments to the Author

The manuscript by Maudult et al describes the creation of 'In situ marine molecule logger' aka I-SMEL, this is a new underwater sampling device that the group has created that pumps seawater across solid phase extraction (SPE) filters. An interesting approach is that the device is portable and can be configured to pump specific volumes and is designed to sit above marine organisms. A strength of this new device is that it does not require the researchers to remove organisms from their native habitat which will greatly aid in studies aimed at longitudinal sampling with minimal impact to the ecosystem. Given the complexities of environmental sampling, this is a well described and thoughtout initial study to pilot the device. The team had three distinct experiments that they carried out: 1) sampling the surrounding water, 2) sampling the area above the sponges compared to small samples of the sponges themselves and finally 3) sampling multiple individual sponges for reproducibility. The combined experiments highlight a number of findings, namely: 1) that sponge specialized metabolites are indeed

secreted into the near water column of the sponge environment, 2) reproducibility of the sponge metabolome across individuals, even within the same environment, is variable, and 3) that metabolomics of the result ocean water filtrates is feasible and a sensitive approach for sampling the environment. This study will likely be impactful across a number of fields including marine chemical ecology, drug discovery, and analytical chemistry. This device could be a real game changer for how we sample the marine environment more reproducibly and rigorously.

I would like to commend the authors for making their data publicly accessible and providing a very thorough SI for the metabolomics data. This is a beautiful use of GNPS and metabolomics tools.

Below please find a list of major and minor critiques for the authors' consideration.

Major

1. The biggest critique of this paper is that researchers will only be able to benefit from this report if they can access the device. This appears to be a homemade device and the construction of the device and specifications are under described. It would be helpful perhaps to include a video as to how to construct the device or include diagrams with part numbers and dimensions so that others can construct the I-SMEL. Additionally, some sort of scale bar would be helpful for Figure 1C to provide a scope for size if one were planning to construct the device. The team mentions being able to change the filters underwater, based on the lack of design details, this reviewer was not able to visualize how that might occur or if there would be other considerations, perhaps having the filters (4, in figure 1) as it's own exploded view would be helpful towards this end.

2. Figure 3. This figure was difficult to interpret in panels B and C. For instance, in B, did the AA-peptides not break down by sponge type? The feature distribution only adds to 84%, are we to infer the other 16% is the crude extract alone? If yes, it does not appear to scale as the other boxes do? Is this also partially confounded by the overlap denoted be *? The sub-breakdown by classes is also difficult to assess since no numbers are assigned to the values, simply colors and box size. Panel C is confusing since this now shows features not % but everything is scaled to the same size overall which doesn't capture the nuances in detection across the different samples, this data may be better served as a table rather than a graphic.

3. The discussion on page 10, lines 164-171 is intriguing. The interindividual variability is fascinating. Could the authors possibly elaborate on detection differences for EX3 further, things that come to mind are what is the half life of these compounds in the water column. Does the type of sponge environmental and time of collection matter, ie if there is a lot of water movement or swell/tidal exchange in a particular environment to they envision this impacting interindividual variability?

Minor

1. I appreciate that the authors display M and RT in figure 4 (also it says Figure 41 in the figured legend) but this is difficult to read and retention time does not add much for thinking about m/z differences in analogues. The addition of RT was distracting and could just appear in the SI tables as they already included.

2. Page 12, line 204 typo tofuranoterpenoïds should probably be 'to furanoterpenoids'

3. Regarding the discussion on Page 16, line 259-260 and the sponge Ems being analogous to flower volatiles, can the authors comment on limits of detection, sampling volume, and the environment? Specifically, it may be that some compounds in the sponge are released into the environment at very low levels and perhaps longer sample or a higher volume of sample to be filtered would be necessary to truly detect more compounds. Much like resins used in culture, is there a possibility that the filters become saturated which may impact limits of detection as well? Basically, how comprehensive would one need to be to fully assess whether a sponge metabolite is retained or excreted at different concentrations.

Reviewer: 3

Comments to the Author

Review of oc-2023-00661c

This is a very high quality and interesting study that describes the development and preliminary testing of a new instrument for non-destructive capture of secondary metabolites from the marine environment. The introduction was particularly well written, and was an enjoyable read. The figures are exemplary, and the quality of the analytical chemistry is first-rate. I reviewed the SI file carefully, and was impressed with the careful annotations, as well as the addition of confidence levels, and full reproduction of the GNPS butterfly plots to standards spectra.

My main criticism of this manuscript is that it does not have a clear focus. The title and the early sections suggest that the goal is to report a new technology (the I-SMEL system). However the paper itself does not discuss the design of this new tool, nor are any details presented about how it is constructed, outside of a small panel in Figure 1 and some paragraphs in the methods section. Therefore, it does not meet the goal of presenting and discussing a new technology. It is also not clear whether this instrument

will be made available to the community, either through the release of plans for fabrication in a future manuscript or through partnership with an instrumentation company.

Much of the body of the manuscript describes the chemical analysis of samples taken using the system, and comparing them to extracts from segments of authentic sponge material. As stated above, the analysis itself is excellent and the experimental design is sound, but many of the subsequent conclusions are quite speculative. I was left wondering what problem the new technology will solve? There were hints of this with the examination of different concentrations of metabolites from sponge material and excreted water column samples, but this could have been influenced by varying levels of adsorption onto the DVB support for different compound classes. Therefore, given that one is limited to collecting three samples per dive, I would have liked to see a stronger demonstration of how this new method will influence the field of marine natural products. The authors did not place much focus on yields of material for this method, but it appears likely that this is a method that will allow mass spectrometry-based analyses rather than NMR structure determination. While this is not an issue if sponge material is used to isolate larger amounts of priority molecules it does detract from the argument that this is more ecologically responsible than collecting and profiling sponge tissue.

I tested the links to all open data. The Zenodo link does not currently work (https://doi.org/10.5281/zenodo.7820941). The GNPS job is available. The MassIVE dataset is currently private and not accessible.

Minor comments:

Figure 1a needs to be improved. The schematic does not really explain how the system is created, and the labels (1 - 5) are not defined in the caption.

There are other papers describing similar technologies, some of which have also studied sponge chemistry. Two examples are (10.1371/journal.pone.0100474) and the very recent preprint (10.1101/2023.03.02.530684).

Figure 3 is very important, but it is quite dense and hard to follow. In particular, the three types of features are not well defined in the images. What do black boxes mean? And white ones? Are identical and related to crude both colored? It is hard to follow the logic here.

What is the rationale for choosing DVB discs? Why this and not a different solid support for this application?

Line 284: 'prodiving' should be 'providing'

Reviewer: 4

Comments to the Author

In “In Situ Capture and Real Time Enrichment of Marine Chemical Diversity” Mauduit and Co-Workers describe a new in situ solid phase extraction device to capture metabolites from seawater. Highlighting the complexities and challenges of identifying molecules present in seawater, the authors argue that there is a general need for capturing and analyzing these molecules for various applications, including marine ecology and drug discovery. To tackle this, the authors developed an instrument named In Situ Marine Molecule Logger or short “I-SMEL” that aims to rapidly capture and enrich specialized exometabolites released by key marine species in their natural environments, which the combined with non-targeted LC-MS/MS analysis and a series of computational data analysis tools.

In general I think there is indeed a great need for new methods to capture the chemotype of complex environments and in situ applications are especially interesting for spatially resolved sampling and to reduce large sample volumes and the paper and developed method should be of broad interest to marine chemical ecologists and natural product chemists. The paper describes mainly the proof-of-concept application of the device and the chemotype in close proximity to marine sponges and compares sponge secreted metabolite levels in sea water to tissue sample and the main novelty is the device in my opinion. I could see this paper also perfectly fitting into an analytical chemistry journal, however, whether it fits the scope of ACS Central Science I would leave up to the editor.

In general, I think the authors did a great job and the paper and the methodology seem solid to me. The only thing the authors might want to discuss a little more, are the extraction efficiency of their method. For global DOM analysis, the highest extractions yields are typically reached by acidifying the water. Is there a possibility to do this with your device, and did you compare how much of an influence this would make?

With regards to your data deposition, its great that you provided the GNPS link and Massive ID. However, please make sure to make the dataset public. As of now I could not access the raw data on MassiVE. I assume it’s still private?

Other minor comments are:

Line 52: “precluding any taxonomical guidance” might be a bit hard wording as there are probably many example where were taxonomical /biosynthetic connection had be established. Consider rephrasing.

Line 66: not sure if “seascapes” is clearly defined term. Perhaps better consider rephrasing. Same for

“ busy highways of putative allelochemicals”. I am not completely sure what you mean here.

Se also that other section in the manuscript line 116, 333, 336, 338 etc.

Line 149: I assume you mean within the molecular network, perhaps specify.

Line 295-297: How do you envision to make your device available to the community?

Line 415: Why did you not use the peak areas from the MZmine output? How does this compare to the results from Compas, and how did you connect feature IDs here?

Author's Response to Peer Review Comments:

IMBE | Unité Mixte de Recherche
Institut Méditerranéen de Biodiversité et d'Ecologie
marine et continentale

Re: Revision of Manuscript oc-2023-00661c

Date: September 13, 2023

Dear Pr.,

Please, find along with this letter the revised manuscript entitled "*In situ Capture and Real Time Enrichment of Marine Chemical Diversity"*, which we hope is now acceptable for publication in ACS Central Science.

On behalf of my team of co-authors, I want to thank you and the four reviewers for the constructive and encouraging comments and advice. We believe all the revisions led to a significantly improved manuscript, answering concerns regarding the accessibility of I-SMEL, compiling additional data in supplementary information, and addressing key questions related to game-changing applications.

The following pages go into the details of the revision, answering point-by-point to the reviewers' comments, and correction we made in the revised version of our manuscript.

If the reviewers would like to access to the MS data on the MassIVE server here are the username = MSV000091465_reviewer, and password = 103_ExoMet. All the data will be made publicly accessible upon acceptation of our manuscript.

Your editorial efforts are much appreciated. Please contact me, if there is any further information that I may provide to you.

Sincerely,

Charlotte Simmler

UMR IMBE | AIX-MARSEILLE UNIVERSITÉ - CNRS - IRD - AVIGNON UNIVERSITÉ
Station Marine d'Endoume, 13007 Marseille
Email : Charlotte.simmler@imbe.fr, Tél : +33 (0)4 13 94 50 49

Responses to the Reviewers' Critique

→ Original critique/comments in Times New Roman black.

→ Action/response in Arial blue.

Reviewer 1

Recommendation: Publish in ACS Central Science after minor revisions noted.

Comments:
In this work, Mauduit et al have developed an instrument to concentrate metabolites excreted by three species of sponges, which are known producers of natural products and applied advanced dereplication strategies to describe the chemical diversity captured using untargeted metabolomics. With the advancement in mass spectrometry instruments and data analytics, development of such methods is significant as it will enable elucidation of chemical crosstalk between life forms in the ocean environments. The introduction is very well written and sets the stage for understanding why the development of this sampling instrument is significant. The study is well designed and executed with appropriate replicates and inclusion of crude extracts of sponge specimen for comparison. The confidence level for annotations is clearly stated and appropriate for this study. Few minor points are listed below which will improve the readability of the manuscript.

1. The authors should revise the first line of the abstract. The phrase: "its related ecosystem functions" appears to not fit in the sentence and can be elaborated upon.

→ Thank you we reformulated the sentence : "Analyzing the chemical composition of seawater ***to understand its influence on*** ecosystem functions is a long-lasting challenge'' and subsequently modified the abstract

2. Line 77 Page 4 (introduction section): "Therefore the idea of alternative SPE approaches." The authors could consider citing "R. N. Tuttle et al., Applied and Environmental Microbiology; 2019: 85, e02830-02818 and the preprint titled "Small Molecule in situ Resin Capture – A Compound First Approach to Natural Product Discovery" by Paul Jensen lab here as well https://doi.org/10.1101/2023.03.02.530684

→ Thank you, yes we should have added those references, now it's done in the revised version of our manuscript. These are indeed really interesting methodes to harvest marine molecules and both studies relate the use of DVB resin to adsorb metabolites, notably microbial ones, directly by contact on marine sediments. The purpose of this device is different than ours focused on seawater relased EMs at distance from the producing organisms. Nevertheless conducting both approaches on benthic organisms could be really interesting as it will lead to a mapping of marine molecules: those remaining in contact with benthic organisms and those emitted at distance.

3. A small panel either in main text figure or as supporting figure to provide visual guide of replication and sample types collected would be useful (description in section titled in situ experiments under results). For example: For EXP-2, it appears that a sample was

collected three times on each specimen, and a total of five specimens of each species were collected. Would this be total of 45 (3*5*3) samples for three species? This would then result in 45/5=9 extracts. Similarly, it appears a total of 15 crude extracts were generated, five for each species and they were pooled resulting in 15/5=3 crude extracts. A visual figure panel will assist in understanding of this scheme.

→ Thank you for your pertinent comment. We agree that the sampling strategy needed to be explained using an illustration for a better understanding. We therefore added a figure panel in supporting information.

Briefly:

For EXP1 and 2: one replicate experiment corresponds to the filtration of a total of 2 L of seawater repeated above either 5 random parts of the ecosystem (EXP1) or 5 different sponge specimens (EXP2). During the sampling all three valves are opened. Therefore, for one replicate a total of 10 L of seawater is passed through 3 SPE disks simultaneously.

The three disks are then eluted individually then pooled to provide an average extract of marine chemical seascape (n= 1, EXP1) or an average extract of sponge EMs (n = 1, EXP2). Both EXP1 and 2 were replicated three times, resulting in 3 replicate extracts for each EXP.

- 1 replicate experiment = one average extract from 3 SPE disks simultaneously from **5 different substrates**. In situ sampling duration 5 x 2 min = sampling volume 10 L (5 x 2 L).
- Total of average chemical seascape extracts = 3
- Total of average sponge EM extracts = 3

For EXP3: one replicate experiment corresponds to the filtration of 10 L of seawater above one sponge individual, on three SPE disks simultaneously. After individual elution of these disks, the solvents are combined to obtain one single extract enriched in individual sponge EMs. EXP3 was replicated three times to obtain 3 individual EM extracts/ sponge species.

- 1 replicate experiment = one average extract from 3 SPE disks simultaneously from **1 single sponge individual**. In situ sampling duration 1 x 10 min = sampling volume 10 L.
- Total of individual sponge EM extracts = 3

4. **Methods section:** “These captures, corresponding to EXP2, were done with all valves opened to obtain three separate DVB filters representing three technical replicates.” Was the data collected separately on these technical replicates? I believe separately and that is why authors have 9 individual extracts (3 for each species). These details are hard to parse out as they are divided between methods and results.

→ In the revised version of our manuscript, we tried to avoid parsing out information between the methods and results sections. We hope that our sampling protocol is now clearer with the additional figure in supporting information. See also our answer above.

5. **Based on Fig. 3B**: It appears that 16% (100- (36+20+10+8) of the features are displayed as a black box, which represents crude extracts. Authors can consider labelling 16% on black box to be consistent.

→ Thank you for your advice, this has been changed now in the revised version of the manuscript.

6. The brief explanation on the choice of divenylbenezene polymer as compared to other chemistries available to adsorb metabolites should be moved from methods to results section.

→ We transferred this information in the result section and added more details in supporting information.

7. **Line 192, Page 11**: "The most polar EM aeroplysinin, was also found to be proportionally 25 times more abundant in EM extract than in crude extracts." The crude extracts were generated using C18-SPE which is a different matrix than the DVB polymer used for enriching EMs from sea water. One is extracted from liquid material (10L) and other from solid material (1 g powder). **While the presence and absence comparisons are straightforward, comparing abundances can be tricky due to differences in sample types and extraction procedures**. These should be clarified as a discussion line while comparing the metabolite abundances between the two Or the authors can simply add a phrase "highlighting enrichment capacity of I-SMEL as they have done elsewhere in the manuscript.

→ Yes, we agree with your pertinent remark and advice. We tempered the sentence in the results section.

Page 11, line 192: *Among them, aeroplysinin-1, the most polar EM, was found in the highest proportion. Such difference could have been due to (1) different SPE extraction procedures between EM and crude extracts (2) higher solubility of aeroplysinin-1 favoring its enrichment from seawater.*

8. Figure 4 is mistakenly labelled as Fig 41

→ Thank you for pointing that out. This has been corrected in the revised version of our manuscript

9. Discussion: Page 16 line 258 "*A possible explanation is that sponges do not*…"
As chemical diversity is detected in enriched samples via I-SMEL, some of the metabolites might not be detected in EMs (below detection limit or ionization repressed due to a co-luting metabolite not present in crude extract). This alternative explanation can also be added as the authors cannot rule out this possibility for a subset of detected features.

→ Absolutely, this is another possibility for not detecting all the metabolites as EMs, in addition to the fact that not all specialized metabolites might be released by the sponges. Therefore, we added such consideration in the discussion.

Page 16:….*Some of them might have remained undetected due to (1) their presence in trace quantities below the detection threshold or (2) potential ionization suppression attributed to residual seasalts.*

Reviewer 2

Recommendation: Publish in ACS Central Science after minor revisions noted.

Comments:
The manuscript by Mauduit et al. describes the creation of 'In situ marine molecule logger'aka I-SMEL, this is a new underwater sampling device that the group has created that pumps seawater across solid phase extraction (SPE) filters. An interesting approach is that the device is portable and can be configured to pump specific volumes and is designed to sit above marine organisms. A strength of this new device is that it does not require the researchers to remove organisms from their native habitat which will greatly aid in studies aimed at longitudinal sampling with minimal impact to the ecosystem. Given the complexities of environmental sampling, this is a well described and thought out initial study to pilot the device.

The team had three distinct experiments that they carried out: 1) sampling the surrounding water, 2) sampling the area above the sponges compared to small samples of the sponges themselves and finally 3) sampling multiple individual sponges for reproducibility.

The combined experiments highlight a number of findings, namely: 1) that sponge specialized metabolites are indeed secreted into the near water column of the sponge environment, 2) reproducibility of the sponge metabolome across individuals, even within the same environment, is variable, and 3) that metabolomics of the result ocean water filtrates is feasible and a sensitive approach for sampling the environment. This study will likely be impactful across a number of fields including marine chemical ecology, drug discovery, and analytical chemistry. **This device could be a real game changer for how we sample the marine environment more reproducibly and rigorously.**

→ Thank you very much!

I would like to commend the authors for making their data publicly accessible and providing a very thorough SI for the metabolomics data. This is a beautiful use of GNPS and metabolomics tools.

→ The data are ready to be made available on MASSive as well as ZENODO as soon as our mansucript is accepted for publications.

Below please find a list of major and minor critiques for the authors' consideration

Major

1. The biggest critique of this paper is that researchers will only be able to benefit from this report **if they can access the device**. This appears to be a homemade device and the construction of the device and specifications are under described. It would be helpful perhaps to include a video as to how to construct the device or include diagrams with part numbers and dimensions so that **others can construct the I-SMEL**. Additionally, some sort of scale bar would be helpful for Figure 1C to provide a scope for size if one were planning to construct the device. The team mentions **being able to change the filters underwater,** based on the lack of design details, this reviewer was not able to visualize

how that might occur or if there would be other considerations, perhaps having the filters (4, in figure 1) as its own exploded view would be helpful towards this end.

This is a really important critique and we agree with you that we should have added more information pertaining the construction and the different parts of the device. We therefore added more explanations information in the manuscript and in the supporting information with an assemblage of detailed pictures.

I-SMEL operates on an inherently simple principle. The instrument is made of six primary components as shown in figure 1 and supporting information. The dimensions of the capture chamber can be adjusted according to the research objectives or the target organism. Similarly, the pertistaltic pump's specificity (e.g flow rate) can be tailored to match the intended purpose. The sizes of the two steel enclosures that house respectively the battery and electronic controller are also contingent upon the desired level of autonomy. These two enclosures, along with the electronic controller, were constructed in-house. The dimensions and working features of I-SMEL were selected based on the average size of our primary biological models (benthic invertebrates such as sponges).

We now hope that with the newly added details, our colleagues either in marine chemical ecology or in natural product chemistry will be able to construct an I-SMEL device of their own. Nevertheless, our plans in the future are to develop new generations of optimized I-SMEL that could be shared or rented between marine researchers according to their needs, and could be deployed simultaneously in different environments. Needless to say that we will need more funding and future international collaborations to reach these goals.

2**. Figure 3.** This figure was difficult to interpret in panels B and C. For instance, in B, did the AA-peptides not break down by sponge type? The feature distribution only adds to 84%, are we to infer the other 16% is the crude extract alone?

Yes, 16% of the features correspond to all sponge crude extracts. We added this information on the new figure 3.

If yes, it does not appear to scale as the other boxes do? Is this also partially confounded by the overlap denoted be *?
The overlap denoted * corresponds to metabolites that are detected both in crude sponge extracts and as EMs. The black box corresponds to sponge metabolites that are only detected in their crude extracts and not as EMs.

The sub-breakdown by classes is also difficult to assess since no numbers are assigned to the values, simply colors and box size. Panel C is confusing since this now shows features not % <u>but everything is scaled to the same size overall which doesn't capture the nuances in detection across the different samples</u>, this data may be better served as a table rather than a graphic.

Thank you for your comments. We understood this figure was not clear enough and therefore, we complemented the data presented in figure 3 by tables in supporting information together with explanations on how this figure was made notably in terms of feature distribution.

3. The discussion on page 10, lines 164-171 is intriguing. The interindividual variability is fascinating. Could the authors possibly elaborate on detection differences for EX3 further, things that come to mind are: what is the half-life of these compounds in the water column? Does the type of sponge environmental and time of collection matter, ie if there is a lot of water movement or swell/tidal exchange in a particular environment to they envision this impacting interindividual variability?

We don't really know the half-life of sponge specialized metabolites in the water column but that would actually be a fascinating area for future research. We can easily hypothesize that some emitted compounds might undergo physico-chemical transformations faster than other.

In the Mediterranean Sea, tides and swells are not very prominent. Regarding a specific sponge species, all samplings took place during the same season (June), with short intervals between the days of sampling. In our study, the interindividual variability did not come from a seasonal effects but rather from the unique filter-feeding & metabolic activities of sponges.

Sponges are active filter-feeders, pumping seawater to uptake feeding particles. The pumping activity varies a lot between individuals (Morganti et al. 2019). Individuals targeted for EXP2-3 were undoubtly of various sizes. Unfortunately, the methods to measure sponge size and/or biomass are available for only a little number of species. In our case, this could be measured only for *Spongia officinalis*. The sponge biomass targeted during EXP2-3 varied between 47 g and 154 g dry weight for this species. For the two other target species, we could not obtain such data, as no method exist to determine the sponge biomass from an estimation of the sponge volume.

Moreover, in a given individual, the pumping rate can also vary within hours, as demonstrated a long time ago by Reiswig (1971) in Marine Biology. Unfortunately, nobody can predict the best moment to get the optimal sponge pumping activity. Also this activity is not synchronous in a population, thus, we may have experimented on individuals that were actually having various pumping rates.

There is also an inherent variability of specialized metabolism from one individual to another within the same species. In the past, our team has also documented such variability in sponge specialized metabolism (Ivanisevic et al. 2011, Reverter et al. 2015 and 2018).

Minor

1. I appreciate that the authors display M and RT in figure 4 (also it says Figure 41 in the figured legend) but this is difficult to read and retention time does not add much for thinking about m/z differences in analogues. The addition of RT was distracting and could just appear in the SI tables as they already included.

→ Thank you for your comment. We changed the figure number accordingly. Nevertheless, we would like to keep the display of M/RT as the retention time is a key information as well for structural dereplication (identification of isomers, adducts and in-source fragments).

2. **Page 12, line 204** typo tofuranoterpenoïds should probably be 'to furanoterpenoids

→ Thank you for pointing that out. This has been corrected in the revised version of our manuscript

3. Regarding **the discussion on Page 16, line 259-260** and the sponge Ems being analogous to flower volatiles, can the authors comment on limits of detection, sampling volume, and the environment? Specifically, it may be that some compounds in the sponge are released into the environment at very low levels and perhaps longer sample or a higher volume of sample to be filtered would be necessary to truly detect more compounds. Much like resins used in culture, is there a possibility that the filters become saturated which may impact limits of detection as well? Basically, how comprehensive would one need to be to fully assess whether a sponge metabolite is retained or excreted at different concentrations.

→ Yes, it is possible that some released metabolites still remain below the detection limits in the enriched samples collected with I-SMEL. By increasing the volume of seawater being filtered and therefore the sampling time, we can expect to accumulate those metabolites on the SPE support.

As per the **saturation of the SPE matrix**, there is a possibility for the DVB disks to become saturated, of course. Reaching a saturation will depend on different parameters such as the turbidity of seawater around the sponges, its pumping/filter-feeding activity, the volume of seawater being filtered. For the experiment presented in this manuscript; we did not reach any saturation with 10 L of filtered seawater distributed on 3 SPE disks (= 333 mL through one single disk x 3). The average volume of water to be filtered on SPE disks or membrane is 1 L in general, but according to the Atlantic SPE disks specifications up to 8 L can be filtered on one disk. In the course of our research, not related to the study presented herein, we filtered up to 4 L of seawater through each disk.

To answer your last question: In order to know whether **a sponge metabolite is retained or excreted intermittently**, we would need repetitive captures on the same organism and with a longer sampling time. By extending the capture duration to more than 10 min (e.g. 30 min/ 30 L) on the same organism, we could undoubtedly enhance the quantity of collected extract while also reducing individual variability (by integrating various phases of the sponge pumping activity from low to high).

Reviewer 3

Recommendation: Does not meet the requirements of publishing in ACS Central Science.

Comments:
Review of oc-2023-00661c
This is a very high quality and interesting study that describes the development and preliminary testing of a new instrument for non-destructive capture of secondary metabolites from the marine environment. The introduction was particularly well written, and was an enjoyable read. The figures are exemplary, and the quality of the analytical chemistry is first-rate. I reviewed the SI file carefully, and was impressed with the careful annotations, as well as the addition of confidence levels, and full reproduction of the GNPS butterfly plots to standards spectra.
Thank you for your encouraging comments.
My main criticism of this manuscript is that it does **not have a clear focus**. The title and the early sections suggest that the goal is to report a new technology (the I-SMEL system). However, the paper itself does not discuss the design of this new tool, nor are any details presented about how it is constructed, outside of a small panel in Figure 1 and some paragraphs in the methods section. **Therefore, it does not meet the goal of presenting and discussing a new technology.**

→ The main focus of the present study is to showcase of I-SMEL, a new type of marine instrument built with simple components, easy to be deployed by divers (i.e. handheld) in its ability to capture seawater diluted molecules and target their producing benthic organisms in various underwater configurations (depths, and topography).

Our study demonstrates that in a very short time, 10 min/ experimental replicate, we were able to identify marine exometabolites produced by targeted benthic organisms (i.e. sponges). I-SMEL is different from the other instruments that have been previously deployed as it can work at different depths (tested down to - 20 meters) and under different configurations (overhang cliffs, roof of sea caves). I-SMEL does not need to stay flat or be maintained on a flat surface to work. **I-SMEL is a handheld instrument and can thus easily travel with scientific divers at different places.** These are key characteristics that differ from the first *in situ* SPE instruments but also, to a certain extent, from the artificial sponge device (La Clair JJ et al. 2014).

Moreover, I-SMEL was designed for **waterborne allelochemicals and other seawater diluted molecules**. The instrument targets molecules in seawater at a minimum distance from a producing organism. To that extent I-SMEL differs from the methods published by the team of Paul R. Jensen (SMIRC technic, by Bogdanov A. and co-workers 2023). Nevertheless, both capture and enrichment technics can be used complementarily. Towards the mapping of marine molecules: those remaining in contact with benthic organisms and those emitted at distance.

It is also not clear whether this instrument will be made available to the community, either through the release of plans for fabrication in a future manuscript or through partnership with an instrumentation company.

We totally understand your point and agree with you that we should have added more technical information pertaining to the construction and the different parts of the device. We therefore added more details in the supporting information and emphasized on the specificity of the device in the results and conclusion sections. See also response to Reviewer 2 **Erreur ! Source du renvoi introuvable.**~~above~~.

Much of the body of the manuscript describes the chemical analysis of samples taken using the system, and comparing them to extracts from segments of authentic sponge material. As stated above, the analysis itself is excellent and the experimental design is sound, but many of the subsequent conclusions are quite speculative. I was left wondering **what problem the new technology will solve**?

The game changing applications are related to marine ecology and chemical ecology with key impacts in natural product chemistry.

As demonstrated herein, I-SMEL enables to capture the chemotype of complex marine/ aquatic environments **without collecting large volume of wate**r (e.g. 10 L/ 10 min), while offering the possibility to sample three replicates per dive. As such, I-SMEL **facilitates *in situ* water sampling for down-stream chemical analyses**. As a handheld instrument, I-SMEL can **be used in different topographical situations** (overhang cliffs, sea cave ceilings) to capture waterborne molecules within a delimited space. I-SMEL helps marine chemical ecologists to **spatially resolve the chemistry of seawater** by targeting well-identified species releasing allelochemicals in the benthos. *In situ* filtration of large volume of seawater with the direct adsorption of molecules on SPE matrices **reduces the risks of physico-chemical transformation of waterborne molecules**, by the time the scientific team reaches the laboratory. I-SMEL will therefore enable to map the chemical seascapes of different marine ecosystems and **to qualitatively and quantitatively** follow the EM production of target marine organisms. Ultimately, this application will lead to a better understanding of chemical/ nutrients exchanges in marine ecosystems.

With I-SMEL, all the steps from water sampling to metabolite enrichment and extraction, down to LC-MS or NMR analyses can, thus, be **standardized** facilitating seasonal and spatial comparisons of seawater chemical composition. Results from such comparative analyses will help natural product researchers to **(1) target the most efficient EM producing marine organisms,** (2) **identify the appropriate periods during which autonomous/continuous EM sampling should be performed to obtain the** extracts in quantities compatible with natural product discovery.

The main key functionalities for I-SMEL are: simplifying in situ water sampling through direct SPE, spatially resolving the chemistry of sea water, targeting keystone benthic species, offering the possibility to standardize seawater sampling, handheld instrument, working in different topographical situations.

Our results and discussion sections have been revised to better emphasize on these key points.

There were hints of this with the examination of different concentrations of metabolites from sponge material and excreted water column samples, but this could have been

influenced by **varying levels of adsorption onto the DVB support for different compound classes.**

→ We agree with your remark please see also our answer below related to the rationale for choosing DVB disks as SPE supports, as well as the answer highlighting the detection of specialized metabolites by NMR.

Therefore, given that one is limited to collecting three samples per dive, I would have liked to see a **stronger demonstration of how this new method will influence the field of marine natural products**. The authors did not place much focus on yields of material for this method, but it appears likely that this is a method that will allow mass **spectrometry-based analyses rather than NMR structure determination**. While this is not an issue if sponge material is used to isolate larger amounts of priority molecules it does detract from the argument that this is more ecologically responsible than collecting and profiling sponge tissue.

→ The data collected in this study with I-SMEL constitute a first step towards undertanding how to improve EMs capture both in terms of duration and amount of water to be sampled, if we were to focus specifically on natural product discovery (from isolation to structural elucidation). In its current form and usage, I-SMEL was not employed to collect molecules for natural product isolation and structure elucidation. A future generation of I-SMEL will be designed to that end.

For now, we only performed 10 min capture each time. With regards to the yields of material, each of these captures afforded in average ~15 mg of EM extract (see added data in supporting information) that still contained substantial amount of salts. Therefore, we calculated that with a cumulative 10 h to 24 h capture, we could gather ~100 mg of desalted EM extract. Altogether such amounts are good starting points for purifying compounds if needed. Nowadays, we can perform NMR structure elucidation with < 1 mg of purified molecule (ex: use of 600 MHz equipped with cryoprobe with samples diluted in ~ 80 µL of deuterated solvent, 2 mm NMR tube, see Mauduit M. et al. 2022). Of course, such 10 - 24 h duration is not compatible with a classic SCUBA dive, and thus, will require the design of autonomous I-SMEL instrument.

We conducted also NMR analyses of our 10-min collected average EM extracts (EXP2, supporting information S7 of our revised manuscript) and were able to clearly detect the signals corresponding to key sponges'specialized metabolites for *Aplysina cavernicola* and *Spongia officinalis* (supporting information S7). This means that it is also possible beside LC-MS to perform NMR analysis of the captured EM extracts in order to record a global molecular fingerprint of the seawater surrounding benthic marine organisms.

Please also take a moment to read our comprehensive response regarding the game-changing applications (above), in line with our revised discussion section of our manuscript.

I tested the links to all open data. The Zenodo link does not currently work (https://doi.org/10.5281/zenodo.7820941). The GNPS job is available. The MassIVE dataset is currently private and not accessible.

→ Yes that is correct. All the data are ready to be released in MassIve and Zenodo but only when the final version of our mansucript is accepted for publication. For sure, they will be made freely available.

Minor comments:

Figure 1a needs to be improved. The schematic does not really explain how the system is created, and the labels (1 - 5) are not defined in the caption. There are other papers describing similar technologies, some of which have also studied sponge chemistry. Two examples are (10.1371/journal.pone.0100474) and the very recent preprint (10.1101/2023.03.02.530684).

→ Thank you for pointing that out. Figure 1 was improved accordingly.

The first article relates the deployment of a pumping system connected to SPE cartridges that can work autonomously and mimic the seawater pumping capacities of sponges to accumulate and thus concentrate dissolved/particulate marine molecules.We should not have missed this reference in our manuscript. This reference is now in the revised version of our manuscript.

As opposed to the artificial sponge instrument, I-SMEL works under different configurations (overhang cliffs, roof of sea caves) at higher hyperbaric pressure and does not need to stay flat or be maintain on a flat surface to work. I**-SMEL is a handheld instrument and can thus easily travel with scientific divers at different places.**

We also added the second article related to the use of resin to adsorb metabolites, notably microbial ones, directly by contact on marine sediments. As such, the purpose of this device is different than ours focused on seawater released EMs at distance from the producing organisms. Nevertheless conducting both approaches on benthic organisms could be really interesting as it will lead to a cartography of marine molecule: those staying in contact with benthic organisms and those emitted and captured at a distance.

Figure 3 is very important, but it is quite dense and hard to follow. In particular, the three types of features are not well defined in the images. What do black boxes mean? And white ones? Are identical and related to crude both colored? It is hard to follow the logic here.

→ Thank you for your comments. We understood this figure was not clear enough. We complemented the data presented in figure 3 by tables in supporting information and added explanations on how this figure was made notably in terms of feature distribution.

What is the rationale for choosing DVB discs? Why this and not a different solid support for this application?

→ **Among the different polymeric solid phases, DVB is the most used to enrich water-soluble natural products** (Berlinck et al. 2021) because it is able to retain structurally diverse dissolved metabolites of wide polarity range. As such, DVB solid phase (Bond-Elut-PPL) is used to capture Dissolved Organic Matter (DOM) from filtered seawater (Dittmar et al. 2008, Petras et al. 2017). DVB resins have also been previously deployed in device aiming at passively sampling microalgae toxins in situ (SPATT Solid Phase Adsorption Toxin Tracking [Roué et al. 2018]). More recently, DVB resin (HP-20) lead to the isolation of metabolites directly enriched in the ecosystem (Bogdanov et al. 2023). Finally, previous work focusing on collecting EMs released by sponge in

aquarium used DVB polymeric SPE (Vlachou et al. 2018). Likewise, in our previous work with *Aplysina cavernicola* maintained in aquarium with found out that DVB resins were able to respectively adsorb and desorb all characteristic bromo-spiro-isoxazoles. For all those reasons we chose DVB as a polymeric phase.

The disks were preferred compared to the cartridges as they offer a larger surface of exchange, allowing faster flow rates to be applied, compatible with shorter sampling time. Also, in general, SPE disks are more appropriate when the water to be filtered is charged with different type of particulates. **The disks were chosen instead of resins** as they are, for now, easier to use and change underwater and more importantly, compatible with a **standardized SPE elution/extraction** in the laboratory.

Thank you for asking this question, we added our explanation in the supporting information of our revised manuscript.

Line 284: 'prodiving' should be 'providing'

→ Thank you for pointing that out. This has been corrected in the revised version of our manuscript

Reviewer 4

Recommendation: Publish in ACS Central Science after minor revisions noted.

Comments:
In "In Situ Capture and Real Time Enrichment of Marine Chemical Diversity" Mauduit and Co-Workers describe a new in situ solid phase extraction device to capture metabolites from seawater. Highlighting the complexities and challenges of identifying molecules present in seawater, the authors argue that there is a general need for capturing and analyzing these molecules for various applications, including marine ecology and drug discovery. To tackle this, the authors developed an instrument named In Situ Marine Molecule Logger or short "I-SMEL" that aims to rapidly capture and enrich specialized exometabolites released by key marine species in their natural environments, which the combined with non-targeted LC-MS/MS analysis and a series of computational data analysis tools.

In general, I think there is indeed a great need for **new methods to capture the chemotype of complex environments and in situ applications are especially interesting for spatially resolved sampling and to reduce large sample volumes** and the paper and developed method should be of broad interest to marine chemical ecologists and natural product chemists.

→ Thank you for your very encouraging comments.

The paper describes mainly the proof-of-concept application of the device and the chemotype in close proximity to marine sponges and compares sponge secreted metabolite levels in seawater to tissue sample and **the main novelty is the device** in my opinion. I could see this paper also perfectly fitting into an analytical chemistry journal, however, whether it fits the scope of ACS Central Science I would leave up to the editor.

In general, I think the authors did a great job and the paper and the methodology seem solid to me. The only thing the authors might want to discuss a little more, are the extraction efficiency of their method. For global **DOM analysis**, the highest extractions yields are typically **reached by acidifying the water**. Is there a possibility to do this with your device, and did you **compare how much of an influence this would make**?

→ For the extraction of sponge specialized EMs, we decided to avoid acidification. The resulting protonation of primary amine and guanidine or 2 amino-imidazole moieties characterizing some specialized metabolites might lead to their elution during the washing step with distilled water, thereby reducing the downstream extraction efficiency. Nevertheless, we are aware that acidification is useful when one wants to remove residual sea salts (carbonate ions, in particular).

Our device does not allow water to be acidified prior to its filtration on the SPE disks, as this is directly performed *in situ*. However, back in the laboratory, acidification can be considered during the automatic elution step designed to remove sea-salt (typically with 0.01 M HCl according to Dittmar T. et al. 2008)

We did not compare how much of an influence this acidification would make on the overall EM composition.

With regards to your data deposition, it is great that you provided the GNPS link and Massive ID. However, please make sure to make the dataset public. As of now I could not access the raw data on MassiVE. I assume it's still private?

→ Yes that is correct. All the data are ready to be released in MassIve and Zenodo but only when the final version of our mansucript is accepted for publication. For sure, they will be made freely available. We also added more details in the published supporting information.

Other minor comments are:
Line 52: "*precluding any taxonomical guidance*" might be a bit hard wording as there are probably many examples where were taxonomical /biosynthetic connection had be established. Consider rephrasing.

→ Yes, thank you. We reformulated the sentence in the revised version with the following: *…. their unknown or multiple biosynthetic origins **impeding** taxonomical guidance for structural assignment…*

Line 66: not sure if "**seascapes**" is clearly defined term. Perhaps better consider rephrasing. Same for "***busy highways of putative allelochemicals***". I am not completely sure what you mean here.
See also that other section in the manuscript line 116, 333, 336, 338 etc.

→ These words draw a parallel with terrestrial environments to depict the intricate chemical composition of seawater. Here are the specifics for their definition, explaining the rationale behind our choice of terminology:

Chemical seascape echoes the widely accepted notion of a chemical landscape in terrestrial environments. It refers to the concept of odor landscapes a recognized phenomenon in chemical ecology, which involves dynamic patterns of chemical signals that are emitted by organisms into their surroundings. These essential "fragrances" (a term less applicable in marine chemical ecology) can carry a wealth of information about the emitting organism's identity, physiological state *etc*… (Finnerty PB et al. 2022 in Bioscience). Often volatile in the terrestrial world, such chemical signals serve as the fundamental cornerstone for all chemical exchanges among organisms, giving rise to invaluable ecosystem benefits like pollination. Similar to the atmosphere, chemical seascapes consist of all the "aromas'' / molecules emitted by living marine organisms. However, they also encompass anthropogenic, synthetic, and xenobiotic pollutants, in addition to other molecules naturally present in the environment but that are not biogenic (Jürgens & Bischoff 2016 in Functional Ecology).

From an ecological perspective, these intricate blends of molecules can be thought of as **"highways"** of chemical cues (which are also referred to as **allelochemicals**). These allelochemicals play a role in shaping biodiversity, and the seascape can be compared to a **bustling highway due to the dense traffic of chemical information** that marine organisms need to interpret.

Line 149: I assume you mean within the molecular network, perhaps specify.
Yes, we modified the title of this section accordingly: "Characterization of EM chemical diversity and variability using feature-based molecular network''.

Line 295-297: How do you envision to make your **device available to the community**?

→ I-SMEL is made of easily accessible and purchasable components; so based on the additional description given in supporting information we hope that marine chemical ecologists and natural product chemists can also develop their own device.

Nevertheless, our plans in the future are to construct new generations of I-SMEL that could be shared or rented between marine researchers according to their needs, and could be deployed simultaneously in different environments. Needless to say that we will need more funding and future international collaborations to reach these goals. For now, we think it is of utmost importance to share our results with technical details on I-SMEL. It is a way, for us, to encourage studies in marine chemical ecology and biodiversity while also moving towards the development of sustainable approach in natural product chemistry.

Line 415: Why did you not use the peak areas from the MZmine output?

→ We did not use peak areas from MZmine output because to be honest we are more used to performing such analysis in Compass Data-Analysis. But, we could have done it with MZmine for sure.

How does this compare to the results **from Compas**, and how did you connect feature IDs here?

→ We used scan# to connect feature IDs (cluster IDs) in MZmine back to their corresponding EIC in Compass Data-Analysis. We also added the information pertaining to the exact *m/z* values and retention time.
